# Environmental degradation amplifies species’ responses to temperature variation in a trophic interaction

**DOI:** 10.1111/1365-2656.13069

**Published:** 2019-08-11

**Authors:** Marianne Mugabo, David Gilljam, Laura Petteway, Chenggui Yuan, Mike S. Fowler, Steven M. Sait

**Affiliations:** ^1^ School of Biology, Faculty of Biological Sciences University of Leeds Leeds UK; ^2^ Dynamic Ecology Group, Department of Biosciences, College of Science Swansea University Swansea UK; ^3^ Maths Department, College of Science Swansea University Swansea UK; ^4^ Present address: Department of Aquatic Resources Swedish University of Agricultural Sciences Öregrund Sweden

**Keywords:** climate change, environmental variation, habitat modification, host–parasitoid, life‐history trajectories, phenological mismatch, population cycles, population dynamics

## Abstract

1. Land‐use and climate change are two of the primary drivers of the current biodiversity crisis. However, we lack understanding of how single‐species and multispecies associations are affected by interactions between multiple environmental stressors.
2. We address this gap by examining how environmental degradation interacts with daily stochastic temperature variation to affect individual life history and population dynamics in a host–parasitoid trophic interaction, using the Indian meal moth, *Plodia interpunctella*, and its parasitoid wasp *Venturia canescens*.
3. We carried out a single‐generation individual life‐history experiment and a multigeneration microcosm experiment during which individuals and microcosms were maintained at a mean temperature of 26°C that was either kept constant or varied stochastically, at four levels of host resource degradation, in the presence or absence of parasitoids.
4. At the individual level, resource degradation increased juvenile development time and decreased adult body size in both species. Parasitoids were more sensitive to temperature variation than their hosts, with a shorter juvenile stage duration than in constant temperatures and a longer adult life span in moderately degraded environments. Resource degradation also altered the host's response to temperature variation, leading to a longer juvenile development time at high resource degradation. At the population level, moderate resource degradation amplified the effects of temperature variation on host and parasitoid populations compared with no or high resource degradation and parasitoid overall abundance was lower in fluctuating temperatures. Top‐down regulation by the parasitoid and bottom‐up regulation driven by resource degradation contributed to more than 50% of host and parasitoid population responses to temperature variation.
5. Our results demonstrate that environmental degradation can strongly affect how species in a trophic interaction respond to short‐term temperature fluctuations through direct and indirect trait‐mediated effects. These effects are driven by species differences in sensitivity to environmental conditions and modulate top‐down (parasitism) and bottom‐up (resource) regulation. This study highlights the need to account for differences in the sensitivity of species’ traits to environmental stressors to understand how interacting species will respond to simultaneous anthropogenic changes.

## Graphical abstract

Species are often exposed simultaneously to multiple anthropogenic stressors, but their impacts have mainly been studied independently and in single species. The authors demonstrate that environmental degradation amplifies species’ responses to fluctuating temperatures by directly altering individual life‐history traits and by indirectly affecting top‐down and bottom‐up regulations in a host‐parasitoid trophic interaction.

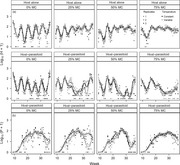

## 1 INTRODUCTION

Anthropogenic environmental modifications through land‐use and climate change are among the primary threats to biodiversity, causing widespread changes in species distribution and species declines and extinctions (Nowakowski, Frishkoff, Agha, Todd, & Scheffers, 2018). Recent climate change, characterized by global warming and changes in the frequency of spatio‐temporal variation in weather conditions (Mann et al., 2017), has induced marked responses across all levels of biological organization, from genes to ecosystems (Scheffers et al., 2016). In particular, short‐term weather fluctuations can affect individual physiological and vital rates, which have considerable impacts on population and community dynamics (reviewed in Vazquez, Gianoli, Morris, & Bozinovic, 2017). Short‐term environmental fluctuations can both stabilize (Estay, Clavijo‐Baquet, Lima, & Bozinovic, 2011) and destabilize (Gonzalez & Descamps‐Julien, 2004) population dynamics depending on the (co‐) variation of species traits (Vazquez et al., 2017) and their intrinsic dynamics (i.e. stable or unstable, Fowler, Laakso, Kaitala, Ruokolainen, & Ranta, 2012). For species in trophic interactions, fluctuating temperatures can have contrasting impacts on species’ life history and population dynamics if they differ in their sensitivity to temperature, through direct and indirect (i.e. via interactions) trait‐mediated effects potentially resulting in phenological mismatches (e.g. Chen, Gols, Biere, & Harvey, 2019). To date, responses of trophic interactions to environmental fluctuations are still poorly understood, but are likely to be complex and difficult to predict (Vazquez et al., 2017).

Anthropogenic habitat modifications can alter eco‐evolutionary dynamics (Alberti, 2015), which affects species assemblages and disrupts ecosystem functions and services (Nichols et al., 2008). Habitat modifications due to land‐use change can restrict species’ access to resources (e.g. habitat fragmentation) and reduce resource quality (e.g. through habitat degradation and agricultural intensification, Fischer & Lindenmayer, 2007). Decreases in resource availability and quality with habitat degradation increase the intensity of intra‐ and interspecific competition for limited resources among primary consumers (e.g. Bostrom‐Einarsson, Bonin, Munday, & Jones, 2013; Bostrom‐Einarsson, Bonin, Munday, & Jones, 2014), which drives changes in species traits (reviewed in Alberti, 2015). The resulting changes in species abundance among primary consumers are passed onto higher trophic levels through species interactions (van der Putten et al., 2004). Differences in responses to anthropogenic stressors between interacting species can lead to phenological mismatches, altering the window of prey vulnerability or resource availability, with the potential to destabilize trophic interactions and food web dynamics (Renner & Zohner, 2018).

Species are often exposed simultaneously to land‐use and climate change, but their impacts have largely been studied independently (Newbold, 2018; Nowakowski et al., 2018). However, recent studies of the cumulative effects of multiple anthropogenic stressors have revealed additive, synergistic and antagonistic effects of land‐use and climate change on demography and abundance (e.g. Newbold, 2018), community composition (e.g. Lindo, Whiteley, & Gonzalez, 2012) and species interactions (e.g. Tylianakis, Didham, Bascompte, & Wardle, 2008). Habitat degradation could have long‐lasting effects on how individuals respond to climate change by affecting life‐history traits, such as development time, body size and body condition (e.g. Jean‐Gagnon et al., 2018). Such changes could have indirect effects on trait‐dependent trophic interactions, such as size‐dependent predation or parasitism in host–parasitoid interactions (Belarde & Railsback, 2016; de Sassi, Staniczenko, & Tylianakis, 2012).

Despite the central role of trophic interactions in community stability and ecosystem functions and services, the way they respond to simultaneous habitat and climate stressors is poorly understood, due to the complexity of direct and indirect effects on species responses. The majority of studies have focused on the effects of increased temperatures or droughts, but few have investigated the effects of weather fluctuations (e.g. Fourcade, Ranius, & Ockinger, 2017; Oliver et al., 2015) and mostly in single species (but see Cardoso, Raffaelli, Lillebo, Verdelhos, & Pardal, 2008). Of these, only one, to our knowledge, investigated the consequences of short‐term temperature fluctuations and their interaction with habitat modification, but only in a single species (Fourcade et al., 2017). These studies show that habitat fragmentation and low patch connectivity limit populations’ capacities to track climatically suitable habitats by reducing dispersal, thus increasing the extinction risk of populations exposed to climate change (e.g. Fourcade et al., 2017; Oliver et al., 2015). However, the interaction between habitat degradation and short‐term environmental fluctuations on life‐history variation and trait‐dependent interactions remains to be investigated in trophically structured systems.

Here, we determined how environmental degradation affected responses to short‐term temperature variation by manipulating host resource quality (plant‐based diet) and daily temperature fluctuations in an insect host–parasitoid interaction. The interaction comprises the moth *Plodia interpunctella* (Pyralidae; Hübner, hereafter “*Plodia*”) and the parasitoid wasp *Venturia canescens* (Ichneumonidae; Gravenhorst, hereafter “*Venturia*”). Their dynamics are characterized by strongly coupled generation cycles in high‐quality environments with constant temperatures (Begon, Sait, & Thompson, 1995; Bjornstad, Sait, Stenseth, Thompson, & Begon, 2001). Resource degradation increases juvenile development time in both species (Boots, 2011; Harvey, Harvey, & Thompson, 1995), which increases the host's window of vulnerability to parasitism (Cronin, Reeve, Xu, Xiao, & Stevens, 2016), and decreases adult fecundity and the intensity of density‐dependent juvenile mortality, which dampens host and parasitoid generation cycles (Cameron, Wearing, Rohani, & Sait, 2007; Knell, Begon, & Thompson, 1998). Juvenile development of both species has similar thermal *optima* and ranges of thermal tolerance, but adult longevity is more sensitive to high temperatures in *Venturia* (Spanoudis & Andreadis, 2012). This model system provides an ideal proxy for trophic interactions in degraded habitats by reproducing dynamics frequently observed in natural populations (e.g. generation cycles, Begon et al., 1995; Bjornstad et al., 2001), which are driven by the same mechanisms involved in species responses to habitat degradation (i.e. variation in food availability, resource quality and intraspecific competition).

We first characterized the effects of resource degradation and temperature variation on host and parasitoid life‐history traits using a single‐generation individual assay of unparasitized and parasitized hosts. We then identified direct and indirect effects through bottom‐up (resource) and top‐down (parasitism) effects on population dynamics using a long‐term microcosm experiment with “host‐alone” (H) and “host–parasitoid” (H‐P) populations. We hypothesized that (a) parasitoid life history will be more affected by temperature variation than the host's through direct effects and (b) resource degradation will have negative effects on host (direct effects) and parasitoid (indirect effects) life history, leading to longer juvenile development time and smaller adult body size in both species. Furthermore, (c) parasitoid life‐history responses to temperature fluctuations should lead to top‐down effects on host and parasitoid dynamics, (d) which should be modulated by resource degradation due to its impact on parasitoid life history. Finally, (e) resource degradation will reduce host and parasitoid abundance and dampen their population cycles through bottom‐up effects and (f) have a stronger impact on host dynamics in “host‐parasitoid” than in “host‐alone” populations through top‐down effects by increasing the host's window of vulnerability to parasitism.

## 2 MATERIALS AND METHODS

### 2.1 Experimental design

We investigated the combined effects of temperature variation and resource degradation at the individual and population levels by carrying out a life‐history assay over a single‐generation and a multigeneration laboratory microcosm experiment for approximately six host generations. Experiments used hosts from laboratory stock cultures and parasitoids from a laboratory parthenogenetic thelytokous (asexual) strain, which were kept on nondegraded host resource at constant 28°C in incubators with a 16:8‐hr light cycle (Sanyo MIR‐553/4, temperature uniformity: ±0.5°C, Panasonic Biomedical).

### 2.2 Temperature variation and resource degradation treatments

Temperature variation and resource degradation were manipulated in a full factorial design. We compared two temperature treatments in each resource degradation treatment. Both temperature treatments were characterized by a mean temperature of 26°C, which did not vary in the *constant* treatment and varied randomly between 22.3 and 30.2°C every 24 hr in the *variable* treatment (26°C ± 1.5°C *SD*, AR(1) = 0.02, 95% CI [−0.10, 0.14]; Figure S1 in Appendix S1). We created a gradient of resource degradation from none to high by replacing 0%, 25%, 50% and 75% of wheat germ in the host's diet (Cameron, Wearing, et al., 2007) with methyl cellulose (MC; Sigma‐Aldrich Company Ltd., CAS no.: 9004‐67‐5, viscosity: 4000 cP). Methyl cellulose is an indigestible bulking agent with no nutritional value (Boots, 2011) and was used as a proxy for reduced plant quality in degraded habitats (e.g. Turlure et al., 2013).

### 2.3 Life‐history assay

The life‐history traits of 40 unparasitized and 25 parasitized individual host larvae (i.e. no competition for resources) were quantified in each temperature and resource degradation treatment. Each host egg was kept individually under its assigned temperature treatment with 0.3 g of its assigned resource (sufficient to reach adult emergence) and monitored daily until death. Host larvae which were to be parasitized were kept in 8 groups of 100 eggs under their assigned temperature treatment with 30.0 g of their assigned resource until they were parasitized. Parasitism took place over a period of three days from age 20 to 22 days (8–9 larva per treatment parasitized on each day as fourth or fifth instars, the most suitable hosts for *Venturia's* development; Harvey, Harvey, & Thompson, 1994). The distinctive ovipositor cocking behaviour following oviposition was used to confirm parasitism (Rogers, 1972). Parasitized larvae were then kept individually under their assigned temperature treatment with 0.3 g of their assigned resource and monitored daily until the death of the host, the parasitoid or both.

We recorded four life‐history traits in non‐parasitized hosts: (a) egg viability, (b) host juvenile stage duration (from egg hatching to adult emergence), (c) adult life span and (d) adult mid‐femur length (which is positively correlated with adult body mass and reproductive fitness; McVean, Sait, Thompson, & Begon, 2002 and references therein). We also recorded four traits in parasitized hosts: (a) proportion of parasitoid encapsulation (i.e. killed by the host's immune system), (b) parasitoid juvenile stage duration (from the day of parasitism to adult emergence), (c) adult life span and (d) adult hind tibia length (which is positively correlated with adult body mass and egg load; Harvey et al., 1994). Legs were measured to the nearest 0.001 mm using a Nikon DS‐5M camera mounted on a Nikon SMZ1500 microscope and the NIS‐element Dv2.3 software (Nikon Instruments).

### 2.4 Population dynamics experiment

Three replicates each of “host‐alone” (H) and “host–parasitoid” (H‐P) microcosms were set up in each temperature by resource treatment, giving a total of 48 (3 × 2 × 2 × 4) microcosms. Each microcosm was established with 15 male and 15 female 5th instar larvae randomly selected from host stock cultures (Begon et al., 1995) in 175 × 116 × 60 mm plastic containers (Azpack) filled with 83 g of the assigned resource. All microcosms were kept at constant 26°C until all 30 host larvae had pupated, after which they were maintained under their assigned temperature treatment (week 1 of the experiment). They were left undisturbed until adult hosts emerged, mated and the first cohort of their offspring had reached the adult stage on week 8. Thereafter, for the remainder of the experiment, one sixth of the resource was replenished sequentially during weekly monitoring by replacing the oldest section with fresh resource of the same treatment (Begon et al., 1995). In H‐P microcosms, the second host cohort was left to develop until fourth and fifth instars were present in all treatments (on week 13). Two newly emerged adult parasitoids randomly selected from stock cultures were then added to each microcosm (Begon et al., 1995). Each week from weeks 9 to 37, dead adults were removed from each microcosm and counted, giving a measure of live host and parasitoid abundance from the previous week (Begon et al., 1995, see raw host and parasitoid time series in Appendix S2).

### 2.5 Statistical analyses

All statistical analyses were carried out in R 3.5.0 (R Core Team 2018).

### 2.6 Life‐history trait analysis

Host and parasitoid juvenile stage duration, adult life span and adult leg length were analysed with linear models using the *gls* procedure of the *nlme* package. Host egg viability was analysed with generalized linear models including a logit link function and binomial error terms. All life‐history models included resource degradation (linear and quadratic terms), temperature treatment and their first‐order interactions as fixed effects. Sex (and its second‐order interactions with resource degradation and temperature treatments) was also included as a fixed effect in the analyses of host juvenile stage duration, adult life span and adult mid‐femur length.

For each trait, an information‐theoretic approach (Burnham & Anderson, 2002) based on Akaike information criterion corrected for small sample size (AIC_c_) was used to compare the full model and all possible combinations of nested models. Supported models (ΔAIC_c_ ≤ 4) were then used to produce estimates of fixed effects and their 95% confidence intervals (CIs). Parameter estimates were obtained from the best supported model (i.e. with the smallest AIC_c_) including that fixed effect instead of using model averaging as averaging models including different contrasts (i.e. different categorical variables) would yield meaningless estimates (Appendix S3). For all linear models, assumptions of residual normality were met (results not shown), but Bartlett's tests revealed significant heteroscedasticity between sexes or treatment groups in some analyses that were accounted for with the *varIdent* function of the *nlme* package.

### 2.7 Time‐series analysis

Average trends in host and parasitoid time series were analysed with generalized additive mixed‐effects models (GAMMs) using the *mgcv*, *gamm4* and *lme4* packages, following Fussmann, Schwarzmuller, Brose, Jousset, and Rall (2014). Negative binomial regressions were used to account for overdispersion in count data (Wood, 2017). First, the parameter *theta* was estimated by running GAMs with the *nb* family (not available in GAMMs), and then, GAMMs were fitted with the *negbin* family, using the *theta* estimates produced by GAMs. Tensor products were used for smooth functions (Wood, 2017). GAMMs included a smooth function of time step for each treatment group (i.e. all possible combinations of temperature, resource degradation and microcosm type), a treatment group intercept, an observer intercept and a microcosm identity random effect. All GAM(M)s were fitted using a Laplace approximation of the maximum likelihood (Wood, 2017).

The cyclical behaviour of host time series was investigated using the *periodogram* procedure of the *TSA* package (Appendix S4). The change in amplitudes over time of normalized adult host time series was analysed following Fussmann et al. (2014). Normalized time series were obtained by dividing the predictions of GAMs by the predictions of generalized linear models with a linear regression for each time series and their amplitudes and the time steps at which they occurred were extracted using the *emd* procedure of the *EMD* package (see Appendix S5 for further details). Parasitoid time series were too short to allow for cycle period and amplitude analyses (Figure S1b in Appendix S2). Normalized amplitude time series were analysed with linear mixed‐effects models using the *lme* procedure. The full model included the experimental week (linear and quadratic terms), experimental treatment (i.e. all possible combinations of temperature, resource degradation and microcosm type) and their first‐order interaction as fixed effects, and microcosm identity as a random effect. We first tested for any temporal correlation of the amplitudes by comparing the independent full model with the autocorrelation full model with a *corAR1* correlation structure. The full *corAR1* model was selected if the correlation structure estimate *Phi* was significantly different from 0 based on 95% CIs. Finally, the mean and variability (measured as the standard deviation (*SD*) of log_10_(N + 1) based on Kendal et al. (1999)) of the overall abundance of dead adult hosts and parasitoids were analysed with linear models using the *gls* procedure of the *nlme* package. All models included resource degradation (linear and quadratic terms), temperature treatment and their first‐order interactions as fixed effects. Models for host data also included microcosm type and its second‐order interactions with resource degradation and temperature treatments. The best supported model(s) were identified by using the same information‐theoretic approach based on AIC_c_ as described above (Appendix S6).

We estimated the relative contribution of direct and indirect effects of resource degradation and temperature variation to host and parasitoid population dynamics by comparing the proportion of null deviance explained by nested models of population data including all subsets of experimental variables and their first‐ and second‐order interactions and estimating their explanatory power (Brooks, Mugabo, Rodgers, Benton, & Ozgul, 2016, see Appendix S7 for further details).

## 3 RESULTS

### 3.1 Life‐history responses

**Figure 1:**
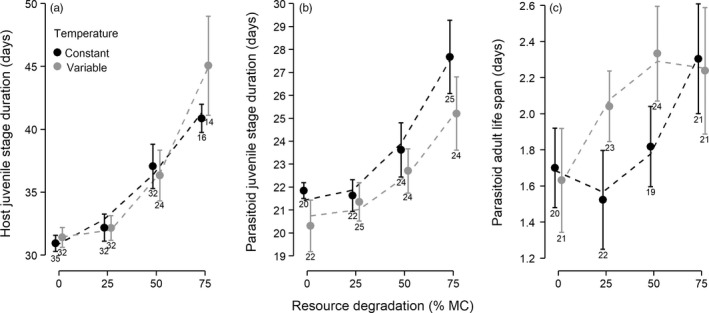
Host and parasitoid life‐history traits were affected by temperature variation and resource degradation (% methyl cellulose). Panels show juvenile stage duration of (a) unparasitized hosts and (b) parasitoids; and (c) parasitoid adult life span. Symbols are observed means and 95% CIs. Regression lines were obtained from the best supported model for each response variable (Tables S2, S4 and S8 in Appendix S3). Numbers below error bars indicate sample size

Host and parasitoid juvenile stage duration increased significantly with resource degradation (Figure 1a,b), and linear and quadratic terms were included in all supported models for both species (Tables S1 and S3 in Appendix S3). Host juvenile stage duration was also significantly affected by an interaction between resource degradation and temperature treatments (linear and quadratic interactions included in 9 and 10 out of 12 supported models, respectively; Table S1 in Appendix S3). This trait was longer under fluctuating than constant temperatures in the most degraded resource treatment (Figure 1a; Table S2 in Appendix S3). However, temperature variation slightly reduced parasitoid juvenile stage duration independently of resource degradation (Figure 1b), with a main negative effect of temperature variation included in all supported models, but only significant in the supported model that did not include interaction terms with resource degradation (interaction terms were not significant, Table S4 in Appendix S3).

Host adult life span was not affected by either treatment (Tables S5 and S6 in Appendix S3). However, parasitoid adult life span was affected by the interaction between resource degradation and temperature variation, with the full model being the only supported model (Table S7 in Appendix S3). Parasitoid adult life span increased more rapidly with resource degradation under variable than constant temperatures (Table S8 in Appendix S3). As a result, adult life span was higher under variable temperatures in low and moderately degraded environments (Figure 1c).

**Figure 2:**
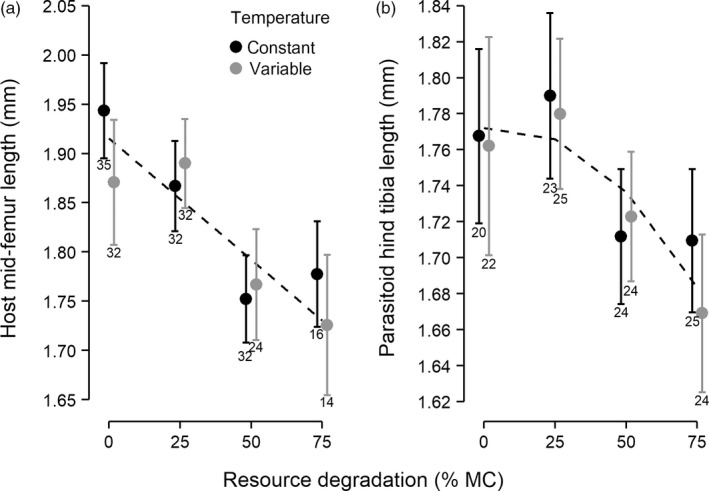
Adult host and parasitoid body sizes were affected by resource degradation, but not by temperature variation. Panels show (a) host mid‐femur length and (b) parasitoid hind tibia length. Symbols are observed means and 95% CIs. The regression lines were obtained from the best supported model for each response variable (Tables S10 and S12 in Appendix S3). Numbers below error bars indicate sample size

Both host and parasitoid adult body size (host mid‐femur and parasitoid hind tibia length) declined significantly with increasing resource degradation, but neither were affected by fluctuating temperatures (Figure 2; Tables S9 and S11 in Appendix S3). Host body size decreased linearly and parasitoid body size decreased quadratically with resource degradation (Figure 2; Tables S10 and S12 in Appendix S3).

Parasitoid encapsulation was low (<8%) and did not substantially vary between treatment groups (0–3 individuals) and was excluded from further analyses. Likewise, life‐history traits of adult hosts which emerged from parasitized larvae were not analysed due to the small sample size (*N*_Total_ = 15). Host egg viability was not significantly affected by either treatment (Tables S13 and S14 in Appendix S3).

### 3.2 Population dynamics

**Figure 3:**
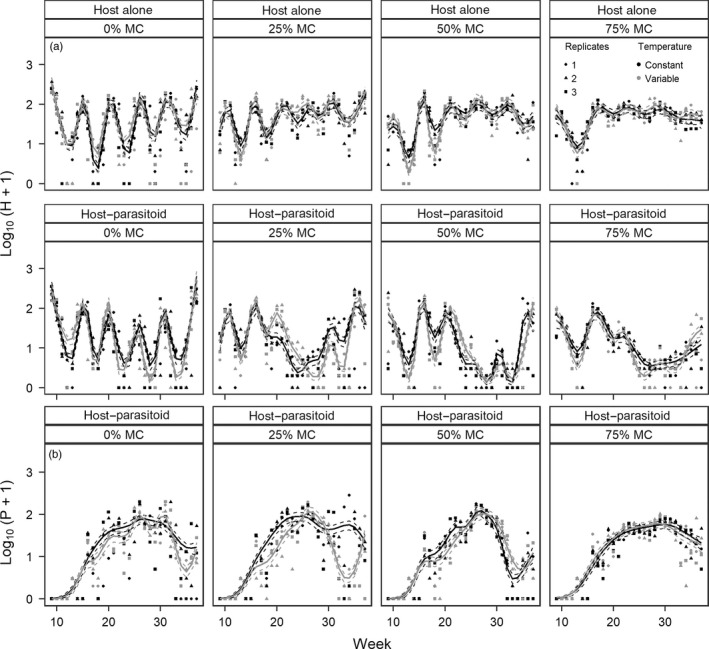
Time series of the number of (a) dead adult hosts (H) and (b) parasitoids (P) according to temperature treatment, resource degradation and microcosm type. Time series were fitted with a GAMM with a negative binomial error distribution and a log link function. GAMM parameters were as follows: number of knots *k* = 14, *theta_host_* = 1.91 and *theta_parasitoid_* = 1.44. Regression lines are GAMM predictions (±*SE*)

The largest differences in host dynamics were observed between H and H‐P microcosms where the presence of parasitoids decreased adult host abundance (Figure 3a; Figure S1a in Appendix S2 for raw time series). Temperature variation had small effects on host dynamics, which differed among levels of resource degradation, with the largest differences observed between constant and variable environments in H‐P microcosms with 25% MC, followed by 0%, 50% and 75% MC and H microcosms with 50% MC (Figure 3a). Host‐alone populations were characterized by generation cycles of a period of one host generation in all but the most degraded resource treatment (75% MC), in which no dominant cycle was detected (Appendix S4). Resource degradation had a dampening effect on host adult cycles, which increased with the level of degradation, causing troughs in abundance to disappear (Figure 3a). Differences in adult abundance were larger between resource treatments in H‐P than in H microcosms (compare top and middle rows in Figure 3a). The more degraded the resource, the more host numbers were suppressed by parasitoids, and for a longer period of time (Figure 3a). Furthermore, generation cycles were not detected in H‐P microcosms, indicating that parasitoids qualitatively altered the host dynamical behaviour under all levels of resource degradation (Appendix S4).

**Figure 4:**
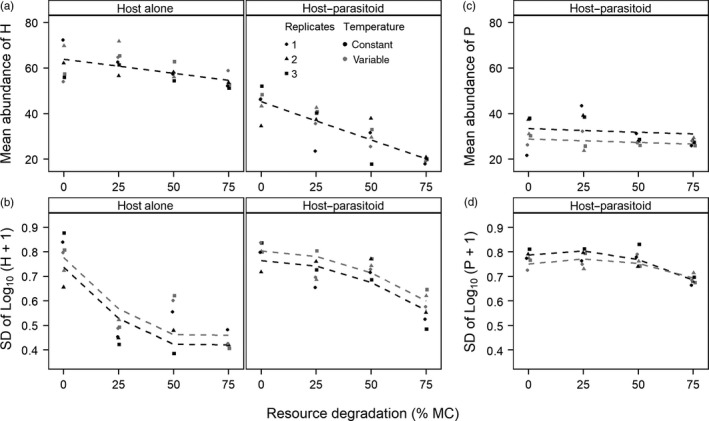
The overall mean and variability of abundance of adult hosts (H; a, b) and parasitoids (P; c, d) were affected by temperature variation, resource degradation and microcosm type (hosts only). Data are observed mean and variability of host and parasitoid abundance. Regression lines were obtained from the best supported models for (a) and (b) (Tables S1 and S3 in Appendix S6), from the second best supported model for (c), which includes a non‐significant effect of resource degradation (ΔAIC_c_ = 1.47; model 6 in Table S5 in Appendix S6), and from the third best supported model for (d), which includes a significant effect of temperature variation (ΔAIC_c_ = 0.62; model 24 in Table S7 in Appendix S6)

The overall mean abundance of dead adult hosts decreased linearly with increasing resource degradation and was lower, with a sharper decrease with resource degradation, in H‐P than in H microcosms (Figure 4a; effects included in all supported models, Tables S1 and S2 in Appendix S6). While mean host abundance was not affected by temperature variation, its effect was included in 5 of the 8 supported models (Figure 4a; Tables S1 and S2 in Appendix S6). However, the variability (*SD*) of host abundance was higher under fluctuating than constant temperatures, decreasing nonlinearly with resource degradation and initially at a faster rate in H than in H‐P microcosms (Figure 4b; effects included in all supported models, Tables S3 and S4 in Appendix S6).

**Figure 5:**
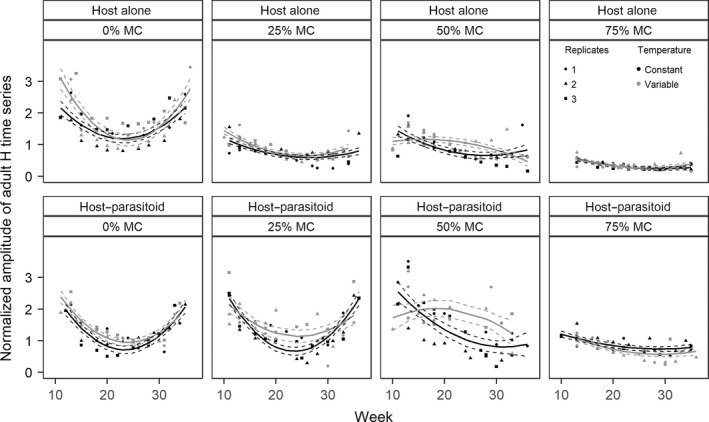
The amplitudes of dead adult host normalized time series were affected by temperature treatments, resource degradation and microcosm types. Data are normalized amplitudes (Appendix S5). Regression lines (±*SE*) were obtained from the only supported model based on AIC_c_

The temporal change in amplitudes of adult host normalized time series was affected by an interaction between time (linear and quadratic terms), temperature, resource degradation and microcosm type (Figure 5; Table S1 in Appendix S5). Amplitude time series were characterized by an intermediate minimum (“U” shape) in all treatment groups except 50% MC under variable temperatures (Table S2 in Appendix S5). Amplitudes initially decreased with time, plateaued around week 25, before increasing towards the end of the experiment (Figure 5). Substantial differences between temperature treatments occurred at all levels of resource degradation except 75% MC. The largest differences occurred between weeks 15 and 30 in H‐P microcosms at 0%, 25% and 50% MC and in H microcosms at 50% MC where amplitudes were higher under variable than constant temperature, with larger differences in more degraded resource treatments (Figure 5). This result means that, during weeks 15–30, the difference between peaks and troughs in host abundance was higher under variable than constant temperatures, and more so in more degraded environments. Finally, changes in amplitude and amplitude variance were higher in H‐P than in H microcosms throughout most of the experiment in all treatment groups except at 0% MC (Figure 5; Table S3 in Appendix S5).

Indirect (top‐down) effects accounted for 21%–59% of the effects of temperature variation on the temporal change, overall mean and variability in host abundance, and change in host normalized amplitudes. They also accounted for 19%–30% of the effects of resource degradation on the same measures (Table S1 and Figure S1 in Appendix S7).

Parasitoid time series were more affected by temperature variation than host populations, but the magnitude of the effects of temperature variation depended on the level of resource degradation (Figure 3b). Parasitoid time series were not affected by temperature variation at 75% MC. At all other levels of resource degradation, parasitoid abundance diverged between temperature treatments from week 18, where abundance was lower under variable temperatures, and most markedly diverged after week 30 when parasitoid abundance declined at a rate depending on the level of resource degradation (Figure 3b). Unlike host dynamics, parasitoid dynamics were not characterized by a marked cyclical behaviour in any of the treatment groups. Instead, parasitoid time series exhibited patterns consistent with multigeneration cycles (Figure 3b; Figure S1b in Appendix S2). Furthermore, at the end of the experiment, two parasitoid populations went extinct, one under variable temperatures with 25% MC and one under constant temperatures with 50% MC (Figure S1b in Appendix S2).

The overall mean of adult parasitoid abundance was significantly affected by temperature treatments, which were included in three of the five supported models (Table S5 in Appendix S6), with 4.5 ± 4.3 (95% CI) fewer individuals under fluctuating than constant temperatures (Figure 4c; Table S6 in Appendix S6). The variance in parasitoid mean abundance was also lower under fluctuating temperatures (Bartlett's test: *K* ^2^ = 6.47, *df* = 1, *p* = .01; Table S6 in Appendix S6). Resource degradation did not significantly affect parasitoid mean abundance (Figure 4c), though its main effect was included in three of the five best supported models and its interaction term with temperature treatments was included in one supported model (Table S5 in Appendix S6). Contrastingly, the variability (*SD*) of adult parasitoid abundance decreased nonlinearly with resource degradation (Figure 4d; Tables S7 and S8 in Appendix S6). A negative effect of temperature variation on the variability of parasitoid abundance was also supported (included in three of the four supported models), but was only significant when a (non‐significant) interaction between temperature treatments and resource degradation was included in the models (Figure 4d; Table S8 in Appendix S6).

Indirect (bottom‐up) effects accounted for at least 56%, 45% and 48% of the effects of temperature variation on the temporal change, overall mean and variability in parasitoid abundance, respectively (Table S2 in Appendix S7).

## 4 DISCUSSION

The mechanisms through which habitat modification, the leading anthropogenic driver of the current biodiversity crisis, can alter the way interacting species respond to environmental variation, are still poorly understood. In particular, how life‐history responses and trait‐dependent species interactions in fluctuating environments are affected by resource degradation (e.g. Turlure et al., 2013) remains to be investigated in trophically structured systems. Using the *Plodia‐Venturia* host–parasitoid model system as a proxy for natural populations in degraded environments, we demonstrated that resource degradation can fundamentally alter species’ responses to daily stochastic temperature fluctuations through both bottom‐up and top‐down effects.

### 4.1 Life‐history responses

In accordance with our predictions, we found that both species were highly sensitive to resource degradation, but that parasitoid life history was more affected by temperature variation than the host's, over a single generation. A small increase in host juvenile stage duration with temperature fluctuations was observed in highly degraded environments, while parasitoid juvenile development was shorter under variable temperatures independently of resource degradation. Short‐term temperature fluctuations that remain within the thermal tolerance range often accelerate juvenile development, but can also slow it in some species, or have no effects in others (reviewed in Colinet, Sinclair, Vernon, & Renault, 2015). Our results suggest that the host and parasitoid species differ in their sensitivity to fluctuating temperatures during the juvenile stage, despite having similar thermal *optima* and ranges of temperature tolerance (Spanoudis & Andreadis, 2012).

Juvenile stage duration increased and adult body size decreased with resource degradation in both species. Such responses were previously observed in response to low host resource nutritional content (Boots, 2011; McVean et al., 2002) and to host resource limitation (Cameron, Metcalfe, Beckerman, & Sait, 2007; Cameron, Wearing, et al., 2007) and are expected to decrease female fecundity and lifetime reproductive success in both species (Harvey et al., 1994; McVean et al., 2002). Resource degradation also modulated the effects of temperature variation on parasitoid adult life span, which increased with host resource degradation and was longer under variable temperatures at moderate (i.e. low and intermediate) levels of resource degradation. Resource limitation has been shown to increase life span in many taxa (reviewed in Szafranski & Mekhail, 2014), while, as with development time, temperature fluctuations have been reported to increase, decrease or to have no effect on adult life span in insects (Colinet et al., 2015). In our experiment, daily temperatures varied between 22.3 and 30.2°C, which exceeds *Venturia's* adult thermal tolerance (Spanoudis & Andreadis, 2012). Thermal acclimation during juvenile development could have increased thermal tolerance at the adult stage by reducing the costs of exposure to harmful temperatures in adults (Colinet et al., 2015; Slotsbo, Schou, Kristensen, Loeschcke, & Sorensen, 2016), but is unlikely to have prolonged adult life span further than under constant temperature as thermal acclimation is energetically costly (Krebs & Feder, 1998).

When exposed to suboptimal temperatures, mobile animals can use behavioural thermoregulation as a buffer, for instance by changing or restricting activity times (Huey et al., 2012). In our experiment, adult parasitoids exposed to temperature variation may have substantially decreased their foraging activity, thus increasing their life span. However, the lack of an effect of temperature variation in nondegraded and highly degraded environments suggests that context‐dependent energetic trade‐offs arose in response to resource limitation and temperature variation. At the highest level of resource degradation, severe resource limitation during juvenile development might have drastically reduced activity in adult parasitoids, promoting longer life span independently of temperature variation. Furthermore, starvation can impair heat tolerance (Mir & Qamar, 2018). Parasitoids that develop in unlimited resources may be able to counteract the energetic costs associated with exposure to suboptimal temperatures and heat tolerance, thus buffering the effects of temperature variation on foraging activity and adult life span (Colinet et al., 2015).

There was no effect of either treatment on parasitoid encapsulation, a measure of the host's immune defences. High temperatures can increase encapsulation by upregulating host defence genes (Seehausen et al., 2017), but species such as *Venturia* limit encapsulation by evading detection by the immune system. This strategy could buffer parasitoids from the indirect effects of environmental change that are mediated through changes in host immunity.

### 4.2 Consequences for host and parasitoid population dynamics

As predicted, we found that parasitoid dynamics were most sensitive to temperature variation, but host resource degradation had complex indirect effects which accounted for nearly 57% of parasitoid population responses to temperature variation. Host dynamics were most affected by resource degradation, which dampened host generation cycles, and by the presence of parasitoids, which exerted strong top‐down regulation. However, host populations also responded to temperature variation and the largest differences between temperature treatments occurred in host–parasitoid populations in which top‐down (indirect) effects contributed to nearly 59% of the effects of temperature variation on the change in host abundance.

In host‐alone populations, transient differences (i.e. lasting for one to two generations) were observed between temperature treatments, most notably at the intermediate level of resource degradation where host cycle amplitudes were larger under variable temperatures. Coupled with our life‐history experiment, these results suggest that exposure to temperature fluctuations within the species’ temperature tolerance range for more than one generation could be necessary to elicit direct life‐history responses in hosts (e.g. Foray, Desouhant, & Gibert, 2014). Surprisingly, host dynamics did not differ between temperature treatments in host‐alone populations maintained at the highest level of resource degradation, despite the stronger sensitivity of juvenile development time to fluctuating temperatures observed under those conditions in our life‐history experiment. In our life‐history assay, unparasitized hosts were kept individually and therefore were not subjected to intraspecific competition for resources. However, intraspecific competition for resources is the main driver of *Plodia's* population dynamics in the absence of parasitoids (e.g. Bjornstad et al., 2001) and can affect species’ responses to temperature variation (i.e. temperature‐dependent competition, e.g. Gonzalez & Descamps‐Julien, 2004; Wilson, Knollenberg, & Fudge, 1984). Therefore, intraspecific competition could have contributed to the response of host‐alone populations to temperature variation, leading to the discrepancy with the results of our life‐history assay.

In host–parasitoid populations, temperature variation had the strongest effects on host and parasitoid dynamics at moderate levels of resource degradation, with marked transient differences between temperature treatments observed in both species. When adult abundance first diverged between temperature treatments, parasitoid abundance was lower and host abundance higher under variable temperatures at all but the highest level of resource degradation. These differences in abundances were most likely due to a lower rate of successful parasitism under variable temperatures rather than differences in host availability, since temperature treatments had a weak effect on host abundance in host‐alone populations. In our life‐history experiment, we showed that temperature variation reduced parasitoid development time independently of resource degradation. This could have led to a phenological mismatch between host and parasitoid populations if most parasitoids emerged before sufficient numbers of suitable hosts were present in the populations (Renner & Zohner, 2018). At the highest levels of resource degradation, host development time was much longer than in other treatment groups, especially under variable temperatures, and fluctuations in host abundance were dampened. A more stable abundance of hosts might therefore have prevented such a phenological mismatch and buffered the effects of temperature variation on host and parasitoid populations. Furthermore, temperature variation increased parasitoid adult life span compared with constant temperatures, but only at moderate levels of resource degradation. A decrease in parasitoid foraging activity in response to temperature fluctuations and host resource degradation could have prolonged adult life span, but would also have decreased parasitoid attack rate (Grigaltchik, Ward, & Seebacher, 2012; Huey et al., 2012).

Overall, fluctuating temperatures led to lower parasitoid abundance and to transient differences in host abundance between temperature treatments at all levels of resource degradation. Transient changes in the intensity of intraspecific competition (Cameron, Metcalfe, et al., 2007; Cameron, Wearing, Rohani, & Sait, 2005), coupled with resource and temperature‐dependent differences in host and parasitoid juvenile development and in parasitoid adult life span, most likely contributed to the complex patterns of population dynamics we observed in this trait‐dependent trophic interaction (Belarde & Railsback, 2016; de Sassi et al., 2012). In koinobiont parasitoids with a shorter window of host suitability than *Venturia* or in idiobiont parasitoids, which only attack discrete host stages (e.g. pupae), such temperature‐dependent differences in host and parasitoid traits are likely to disrupt the phenology of trophic interactions even more than observed here, especially in degraded environments where parasitoid attack rate might be reduced by resource limitation.

In the absence of parasitoids, host dynamics were characterized by generation cycles, which were strongly dampened by resource degradation. In *Plodia*, generation cycles are driven by cycles in the larval stage structure due to asymmetric competition between early and late instars and cannibalism, which lead to density‐dependent mortality in eggs and early instars (Begon et al., 1995; Bjornstad et al., 2001; Sait, Liu, Thompson, Godfray, & Begon, 2000). The dampening effects of resource degradation on host generation cycles are due to the reduction in adult fecundity and of the intensity of density‐dependent juvenile mortality (Cameron, Wearing, et al., 2007; Knell et al., 1998). However, contrary to our predictions and to these previous studies, generation cycles were not detected in either host or parasitoid dynamics in the presence of parasitoids. The relatively short length of host time series combined with the strong top‐down regulation of host abundance might have prevented the statistical detection of generation cycles, despite an apparent cyclical behaviour (Figure 3). The small decrease in the variability of parasitoid abundance suggests that parasitoid dynamics were also dampened by resource degradation, but to a lesser extent than host dynamics. However, unlike the host, there were no clear parasitoid cycles, suggesting that host and parasitoid dynamics were uncoupled, regardless of resource degradation or temperature variation. Instead, parasitoid time series exhibited patterns consistent with multigeneration cycles, but were too short to statistically estimate their cycle period or synchrony with host populations. This potential decoupling of host and parasitoid dynamics may have contributed to the extinction of two parasitoid populations at moderate levels of resource degradation.

The variability of host overall abundance was higher under variable compared with constant temperatures. The amplitude of host cycles was also largely higher under variable temperatures in the presence of parasitoids at all but the highest level of resource degradation, and indirect effects accounted for nearly 35% of variability in host abundance and 25% of cycle amplitudes in fluctuating temperatures. Temperature variation thus had direct and indirect destabilizing effects on host dynamics through top‐down and bottom‐up regulation, which were modulated by the level of resource degradation. Short‐term temperature fluctuations and stochastic environmental variation in general can stabilize or destabilize population dynamics, with equally contrasting effects at the community level (e.g. Estay et al., 2011; Fowler et al., 2012; Fowler & Ruokolainen, 2013; Gonzalez & Descamps‐Julien, 2004). Here, fluctuating temperatures are likely to increase parasitoid extinction risk by increasing host cycle amplitudes and variability in abundance, especially since host and parasitoid populations were not strongly coupled, with greater long‐term risks of pest (host) outbreaks (Sait et al., 2000).

Finally, in accordance with our predictions, we found that the impact of parasitoids on host dynamics increased with the level of resource degradation. The more degraded the resource, the more host abundance was suppressed by parasitoids and top‐down regulation accounted for up to 30% of host population responses to resource degradation. Changes in host cycle amplitude across time were also larger in the presence of parasitoids and more so at moderate levels of resource degradation. Previous experiments in this system reported that parasitoids consistently reduce host abundance and increase the amplitude of fluctuations (e.g. Begon, Sait, & Thompson, 1996); however, no previous work has investigated the impact of host resource degradation. We predicted that host resource degradation would increase the window of vulnerability of the host to parasitism by increasing their development time, which can lead to a larger proportion of parasitized larvae (Cronin et al., 2016). Testing the hypothesis of slow‐growth–high mortality in a host–parasitoid system, Benrey and Denno (1997) showed that low host diet quality was associated with longer development times and higher rates of parasitism. Our results are consistent with this hypothesis and suggest that resource degradation should increase the risk of host and parasitoid extinction by increasing the host's susceptibility to parasitism in addition to the potential decoupling of their dynamics.

## 5 CONCLUSIONS

We demonstrated that resource degradation can amplify species' responses to short‐term stochastic temperature variation by directly altering the quality of individuals through complex patterns of life‐history variation and by indirectly affecting the intensity of top‐down and bottom‐up regulation. Our study highlights the need to account for individual species traits and differences in their sensitivity to multiple environmental stressors in order to understand and predict how trophically interacting species will respond to simultaneous anthropogenic changes.

## AUTHORS' CONTRIBUTIONS

M.M., S.M.S., L.H.P., M.S.F. and C.Y. designed the research. M.M. and L.H.P. carried out the experiments and M.M. analysed the data. M.M., S.M.S., D.G. and M.S.F. wrote the manuscript, and all authors gave their approval before submission for publication.

## Data Availability

Data presented in this manuscript are available at: https://doi.org/10.5285/52b6b432-8f69-4b08-8a47-04e40f656f55 and https://doi.org/10.5285/54b722a7-7cda-4817-86a5-b96ad0bb4ae7 (Mugabo et al., 2019a, 2019b).

## References

[jane13069-bib-0001] Alberti, M. (2015). Eco‐evolutionary dynamics in an urbanizing planet. Trends in Ecology & Evolution, 30, 114–126. 10.1016/j.tree.2014.11.007 25498964

[jane13069-bib-0002] Begon, M. , Sait, S. M. , & Thompson, D. J. (1995) Persistence of a parasitoid‐host system – Refuges and generation cycles. Proceedings of the Royal Society B‐Biological Sciences, 260, 131–137.

[jane13069-bib-0003] Begon, M. , Sait, S. M. , & Thompson, D. J. (1996). Predator‐prey cycles with period shifts between two‐ and three‐species systems. Nature, 381, 311–315. 10.1038/381311a0

[jane13069-bib-0004] Belarde, T. A. , & Railsback, S. F. (2016). New predictions from old theory: Emergent effects of multiple stressors in a model of piscivorous fish. Ecological Modelling, 326, 54–62. 10.1016/j.ecolmodel.2015.07.012

[jane13069-bib-0005] Benrey, B. , & Denno, R. F. (1997). The slow‐growth‐high‐mortality hypothesis: A test using the cabbage butterfly. Ecology, 78, 987–999.

[jane13069-bib-0006] Bjornstad, O. N. , Sait, S. M. , Stenseth, N. C. , Thompson, D. J. , & Begon, M. (2001). The impact of specialized enemies on the dimensionality of host dynamics. Nature, 409, 1001–1006. 10.1038/35059003 11234001

[jane13069-bib-0007] Boots, M. (2011). The evolution of resistance to a parasite is determined by resources. The American Naturalist, 178, 214–220. 10.1086/660833 21750385

[jane13069-bib-0008] Bostrom‐Einarsson, L. , Bonin, M. C. , Munday, P. L. , & Jones, G. P. (2013). Strong intraspecific competition and habitat selectivity influence abundance of a coral‐dwelling damselfish. Journal of Experimental Marine Biology and Ecology, 448, 85–92. 10.1016/j.jembe.2013.06.017

[jane13069-bib-0009] Bostrom‐Einarsson, L. , Bonin, M. C. , Munday, P. L. , & Jones, G. P. (2014). Habitat degradation modifies the strength of interspecific competition in coral dwelling damselfishes. Ecology, 95, 3056–3067. 10.1890/13-1345.1

[jane13069-bib-0010] Brooks, M. E. , Mugabo, M. , Rodgers, G. M. , Benton, T. G. , & Ozgul, A. (2016). How well can body size represent effects of the environment on demographic rates? Disentangling correlated explanatory variables. Journal of Animal Ecology, 85, 318–328. 10.1111/1365-2656.12465 26620593

[jane13069-bib-0011] Burnham, K. P. , & Anderson, D. R. (2002). Model selection and multimodel inference. A practical information‐theoretic approach. New York, NY: Springer.

[jane13069-bib-0012] Cameron, T. C. , Metcalfe, D. , Beckerman, A. P. , & Sait, S. M. (2007). Intraspecific competition: The role of lags between attack and death in host‐parasitoid interactions. Ecology, 88, 1225–1231. 10.1890/06-0661 17536408

[jane13069-bib-0013] Cameron, T. C. , Wearing, H. J. , Rohani, P. , & Sait, S. M. (2005). A koinobiont parasitoid mediates competition and generates additive mortality in healthy host populations. Oikos, 110, 620–628. 10.1111/j.0030-1299.2005.13964.x

[jane13069-bib-0014] Cameron, T. C. , Wearing, H. J. , Rohani, P. , & Sait, S. M. (2007). Two‐species asymmetric competition: Effects of age structure on intra‐ and interspecific interactions. Journal of Animal Ecology, 76, 83–93. 10.1111/j.1365-2656.2006.01185.x 17184356

[jane13069-bib-0015] Cardoso, P. G. , Raffaelli, D. , Lillebo, A. I. , Verdelhos, T. , & Pardal, M. A. (2008). The impact of extreme flooding events and anthropogenic stressors on the macrobenthic communities' dynamics. Estuarine Coastal and Shelf Science, 76, 553–565. 10.1016/j.ecss.2007.07.026

[jane13069-bib-0016] Chen, C. , Gols, R. , Biere, A. , & Harvey, J. A. (2019). Differential effects of climate warming on reproduction and functional responses on insects in the fourth trophic level. Functional Ecology, 33, 693–702. 10.1111/1365-2435.13277

[jane13069-bib-0017] Colinet, H. , Sinclair, B. J. , Vernon, P. , & Renault, D. (2015). Insects in fluctuating thermal environments. Annual Review of Entomology, 60, 123–140. 10.1146/annurev-ento-010814-021017 25341105

[jane13069-bib-0018] Cronin, J. T. , Reeve, J. D. , Xu, D. S. , Xiao, M. Q. , & Stevens, H. N. (2016). Variable prey development time suppresses predator‐prey cycles and enhances stability. Ecology Letters, 19, 318–327. 10.1111/ele.12571 26778037

[jane13069-bib-0019] de Sassi, C. , Staniczenko, P. P. A. , & Tylianakis, J. M. (2012). Warming and nitrogen affect size structuring and density dependence in a host‐parasitoid food web. Philosophical Transactions of the Royal Society B‐Biological Sciences, 367, 3033–3041. 10.1098/rstb.2012.0233 PMC3479742 23007092

[jane13069-bib-0020] Estay, S. A. , Clavijo‐Baquet, S. , Lima, M. , & Bozinovic, F. (2011). Beyond average: An experimental test of temperature variability on the population dynamics of *Tribolium confusum* . Population Ecology, 53, 53–58. 10.1007/s10144-010-0216-7

[jane13069-bib-0021] Fischer, J. , & Lindenmayer, D. B. (2007). Landscape modification and habitat fragmentation: A synthesis. Global Ecology and Biogeography, 16, 265–280. 10.1111/j.1466-8238.2007.00287.x

[jane13069-bib-0022] Foray, V. , Desouhant, E. , & Gibert, P. (2014). The impact of thermal fluctuations on reaction norms in specialist and generalist parasitic wasps. Functional Ecology, 28, 411–423. 10.1111/1365-2435.12171

[jane13069-bib-0023] Fourcade, Y. , Ranius, T. , & Ockinger, E. (2017). Temperature drives abundance fluctuations, but spatial dynamics is constrained by landscape configuration: Implications for climate‐driven range shift in a butterfly. Journal of Animal Ecology, 86, 1339–1351. 10.1111/1365-2656.12740 28796909

[jane13069-bib-0024] Fowler, M. S. , Laakso, J. , Kaitala, V. , Ruokolainen, L. , & Ranta, E. (2012). Species dynamics alter community diversity‐biomass stability relationships. Ecology Letters, 15, 1387–1396. 10.1111/j.1461-0248.2012.01862.x 22931046

[jane13069-bib-0025] Fowler, M. S. , & Ruokolainen, L. (2013). Colonization, covariance and colour: Environmental and ecological drivers of diversity‐stability relationships. Journal of Theoretical Biology, 324, 32–41. 10.1016/j.jtbi.2013.01.016 23416170

[jane13069-bib-0026] Fussmann, K. E. , Schwarzmuller, F. , Brose, U. , Jousset, A. , & Rall, B. C. (2014). Ecological stability in response to warming. Nature Climate Change, 4, 206–210. 10.1038/nclimate2134

[jane13069-bib-0027] Gonzalez, A. , & Descamps‐Julien, B. (2004). Population and community variability in randomly fluctuating environments. Oikos, 106, 105–116. 10.1111/j.0030-1299.2004.12925.x

[jane13069-bib-0028] Grigaltchik, V. S. , Ward, A. J. W. , & Seebacher, F. (2012). Thermal acclimation of interactions: Differential responses to temperature change alter predator‐prey relationship. Proceedings of the Royal Society B‐Biological Sciences, 279, 4058–4064. 10.1098/rspb.2012.1277 PMC3427582 22859598

[jane13069-bib-0029] Harvey, J. A. , Harvey, I. F. , & Thompson, D. J. (1994). Flexible larval growth allows use of a range of host sizes by a parasitoid wasp. Ecology, 75, 1420–1428. 10.2307/1937465

[jane13069-bib-0030] Harvey, J. A. , Harvey, I. F. , & Thompson, D. J. (1995). The effect of host nutrition on growth and development of the parasitoid wasp *Venturia canescens* . Entomologia Experimentalis Et Applicata, 75, 213–220. 10.1111/j.1570-7458.1995.tb01929.x

[jane13069-bib-0031] Huey, R. B. , Kearney, M. R. , Krockenberger, A. , Holtum, J. A. M. , Jess, M. , & Williams, S. E. (2012). Predicting organismal vulnerability to climate warming: Roles of behaviour, physiology and adaptation. Philosophical Transactions of the Royal Society B‐Biological Sciences, 367, 1665–1679. 10.1098/rstb.2012.0005 PMC3350654 22566674

[jane13069-bib-0032] Jean‐Gagnon, F. , Legagneux, P. , Gilchrist, G. , Belanger, S. , Love, O. , & Bety, J. (2018). The impact of sea ice conditions on breeding decisions is modulated by body condition in an arctic partial capital breeder. Oecologia, 186, 1–10. 10.1007/s00442-017-4002-5 29143150

[jane13069-bib-0033] Kendall, B. E. , Briggs, C. J. , Murdoch, W. W. , Turchin, P. , Ellner, S. P. , McCauley, E. , … Wood, S. N. (1999). Why do populations cycle? A synthesis of statistical and mechanistic modeling approaches. Ecology, 80, 1789–1805. 10.1890/0012-9658(1999)080[1789:WDPCAS]2.0.CO;2

[jane13069-bib-0034] Knell, R. J. , Begon, M. , & Thompson, D. J. (1998). Host‐pathogen population dynamics, basic reproductive rates and threshold densities. Oikos, 81, 299–308. 10.2307/3547050

[jane13069-bib-0035] Krebs, R. A. , & Feder, M. E. (1998). Experimental manipulation of the cost of thermal acclimation in *Drosophila melanogaster* . Biological Journal of the Linnean Society, 63, 593–601. 10.1111/j.1095-8312.1998.tb00331.x

[jane13069-bib-0036] Lindo, Z. , Whiteley, J. , & Gonzalez, A. (2012). Traits explain community disassembly and trophic contraction following experimental environmental change. Global Change Biology, 18, 2448–2457. 10.1111/j.1365-2486.2012.02725.x

[jane13069-bib-0037] Mann, M. E. , Rahmstorf, S. , Kornhuber, K. , Steinman, B. A. , Miller, S. K. , & Coumou, D. (2017). Influence of anthropogenic climate change on planetary wave resonance and extreme weather events. Scientific Reports, 7, 45242. 10.1038/srep45242 PMC5366916 28345645

[jane13069-bib-0038] McVean, R. I. K. , Sait, S. M. , Thompson, D. J. , & Begon, M. (2002). Effects of resource quality on the population dynamics of the Indian meal moth *Plodia interpunctella* and its granulovirus. Oecologia, 131, 71–78. 28547512 10.1007/s00442-001-0862-8

[jane13069-bib-0039] Mir, A. H. , & Qamar, A. (2018). Effects of starvation and thermal stress on the thermal tolerance of silkworm, *Bombyx mori*: Existence of trade‐offs and cross‐tolerances. Neotropical Entomology, 47, 610–618. 10.1007/s13744-017-0559-2 28956278

[jane13069-bib-0040] Mugabo, M. , Gilljam, D. , Petteway, L. , Yuan, C. , Fowler, M. S. , & Sait, S. M. (2019a). Individual life history data from a resource degradation and temperature variation life history experiment in the Plodia‐Venturia host‐parasitoid interaction. Lancaster, UK:NERC Environmental Information Data Centre.

[jane13069-bib-0041] Mugabo, M. , Gilljam, D. , Petteway, L. , Yuan, C. , Fowler, M. S. , & Sait, S. M. (2019b). Population count data from a resource degradation and temperature variation population dynamics experiment in the Plodia‐Venturia host‐parasitoid interaction. Lancaster, UK:NERC Environmental Information Data Centre.

[jane13069-bib-0042] Newbold, T. (2018). Future effects of climate and land‐use change on terrestrial vertebrate community diversity under different scenarios. Proceedings of the Royal Society B‐Biological Sciences, 285, 20180792. 10.1098/rspb.2018.0792 PMC6030534 29925617

[jane13069-bib-0043] Nichols, E. , Spector, S. , Louzada, J. , Larsen, T. , Amequita, S. , Favila, M. E. , & Scarabaeinae Res, N. (2008). Ecological functions and ecosystem services provided by Scarabaeinae dung beetles. Biological Conservation, 141, 1461–1474. 10.1016/j.biocon.2008.04.011

[jane13069-bib-0044] Nowakowski, A. J. , Frishkoff, L. O. , Agha, M. , Todd, B. D. , & Scheffers, B. R. (2018). Changing thermal landscapes: Merging climate science and landscape ecology through thermal biology. Current Landscape Ecology Reports, 3, 57–72. 10.1007/s40823-018-0034-8

[jane13069-bib-0045] Oliver, T. H. , Marshall, H. H. , Morecroft, M. D. , Brereton, T. , Prudhomme, C. , & Huntingford, C. (2015). Interacting effects of climate change and habitat fragmentation on drought‐sensitive butterflies. Nature Climate Change, 5, 941–945. 10.1038/nclimate2746

[jane13069-bib-0060] R Core Team . (2018). R: A language and environment for statistical computing. Vienna, Austria:R Foundation for Statistical Computing https://www.R-project.org/

[jane13069-bib-0046] Renner, S. S. , & Zohner, C. M. (2018). Climate change and phenological mismatch in trophic interactions among plants, insects, and vertebrates. Annual Review of Ecology, Evolution, and Systematics, 49, 165–182. 10.1146/annurev-ecolsys-110617-062535

[jane13069-bib-0047] Rogers, D. (1972). Ichneumon wasp *Venturia canescens* – Oviposition and avoidance of superparasitism. Entomologia Experimentalis Et Applicata, 15, 190–194.

[jane13069-bib-0048] Sait, S. M. , Liu, W. C. , Thompson, D. J. , Godfray, H. C. J. , & Begon, M. (2000). Invasion sequence affects predator‐prey dynamics in a multi‐species interaction. Nature, 405, 448–450. 10.1038/35013045 10839538

[jane13069-bib-0049] Scheffers, B. R. , De Meester, L. , Bridge, T. C. L. , Hoffmann, A. A. , Pandolfi, J. M. , Corlett, R. T. , … Watson, J. E. M. (2016). The broad footprint of climate change from genes to biomes to people. Science, 354, aaf7671 10.1126/science.aaf7671 27846577

[jane13069-bib-0050] Seehausen, M. L. , Cusson, M. , Regniere, J. , Bory, M. , Stewart, D. , Djoumad, A. , … Martel, V. (2017). High temperature induces downregulation of polydnavirus gene transcription in lepidopteran host and enhances accumulation of host immunity gene transcripts. Journal of Insect Physiology, 98, 126–133. 10.1016/j.jinsphys.2016.12.008 28041943

[jane13069-bib-0051] Slotsbo, S. , Schou, M. F. , Kristensen, T. N. , Loeschcke, V. , & Sorensen, J. G. (2016). Reversibility of developmental heat and cold plasticity is asymmetric and has long‐lasting consequences for adult thermal tolerance. Journal of Experimental Biology, 219, 2726–2732. 10.1242/jeb.143750 27353229

[jane13069-bib-0052] Spanoudis, C. G. , & Andreadis, S. S. (2012). Temperature‐dependent survival, development, and adult longevity of the koinobiont endoparasitoid *Venturia canescens* (Hymenoptera: Ichneumonidae) parasitizing *Plodia interpunctella* (Lepidoptera: Pyralidae). Journal of Pest Science, 85, 75–80. 10.1007/s10340-011-0405-y

[jane13069-bib-0053] Szafranski, K. , & Mekhail, K. (2014). The fine line between lifespan extension and shortening in response to caloric restriction. Nucleus, 5, 56–65. 10.4161/nucl.27929 24637399 PMC4028356

[jane13069-bib-0054] Turlure, C. , Radchuk, V. , Baguette, M. , Meijrink, M. , van den Burg, A. , WallisDeVries, M. , & van Duinen, G. J. (2013). Plant quality and local adaptation undermine relocation in a bog specialist butterfly. Ecology and Evolution, 3, 244–254. 10.1002/ece3.427 23467336 PMC3586634

[jane13069-bib-0055] Tylianakis, J. M. , Didham, R. K. , Bascompte, J. , & Wardle, D. A. (2008). Global change and species interactions in terrestrial ecosystems. Ecology Letters, 11, 1351–1363. 10.1111/j.1461-0248.2008.01250.x 19062363

[jane13069-bib-0056] van der Putten, W. H. , de Ruiter, P. C. , Bezemer, T. M. , Harvey, J. A. , Wassen, M. , & Wolters, V. (2004). Trophic interactions in a changing world. Basic and Applied Ecology, 5, 487–494. 10.1016/j.baae.2004.09.003

[jane13069-bib-0057] Vazquez, D. P. , Gianoli, E. , Morris, W. F. , & Bozinovic, F. (2017). Ecological and evolutionary impacts of changing climatic variability. Biological Reviews, 92, 22–42. 10.1111/brv.12216 26290132

[jane13069-bib-0058] Wilson, D. S. , Knollenberg, W. G. , & Fudge, J. (1984). Species packing and temperature‐dependent competition among burying beetles (Silphidae, *Nicrophorus*). Ecological Entomology, 9, 205–216.

[jane13069-bib-0059] Wood, S. N. (2017). Generalized additive models: An introduction with R (Second edition). Taylor and Francis.

