# Supplementary material for: Environmental degradation amplifies species’ responses to temperature variation in a trophic interaction
Source: J Anim Ecol. 2019 Aug 11;88(11):1657–69. doi: 10.1111/1365-2656.13069 (PMC6899768; doi:10.1111/1365-2656.13069)
Supplement: Supplementary file 1 [file JANE-88-1657-s001.docx]

# Appendix S1 – Variable temperature time series

**
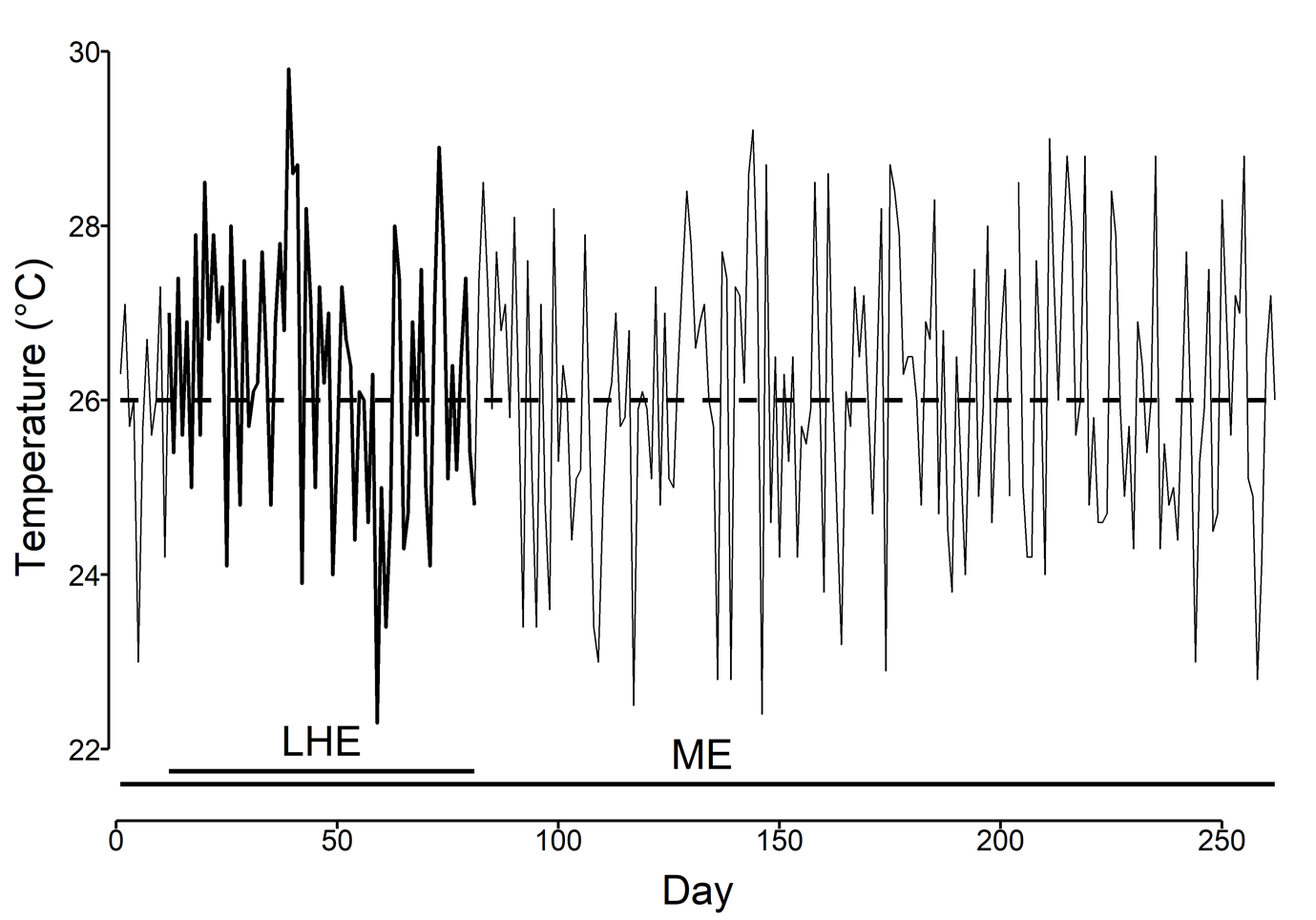
**

**Figure S1.** Temperature time series in the constant (dashed line) and variable (solid line) treatments. The thicker part of the time series represents the period during which the life history experiment (LHE) was conducted. The life history experiment (LHE, mean = 26.29 ºC, SD = 1.48 ºC, range = [22.3, 29.8 ºC], AC = 0.05 with 95% CI [-0.19, 0.28] lasted for 70 days and the microcosm experiment (ME, mean = 26.13 ºC, SD = 1.54 ºC, range = [22.3, 30.2 ºC], AC = 0.02 with 95% CI [-0.10, 0.14]) for 262 days.

# Appendix S2 – Raw time series of the number of dead adult hosts and parasitoids


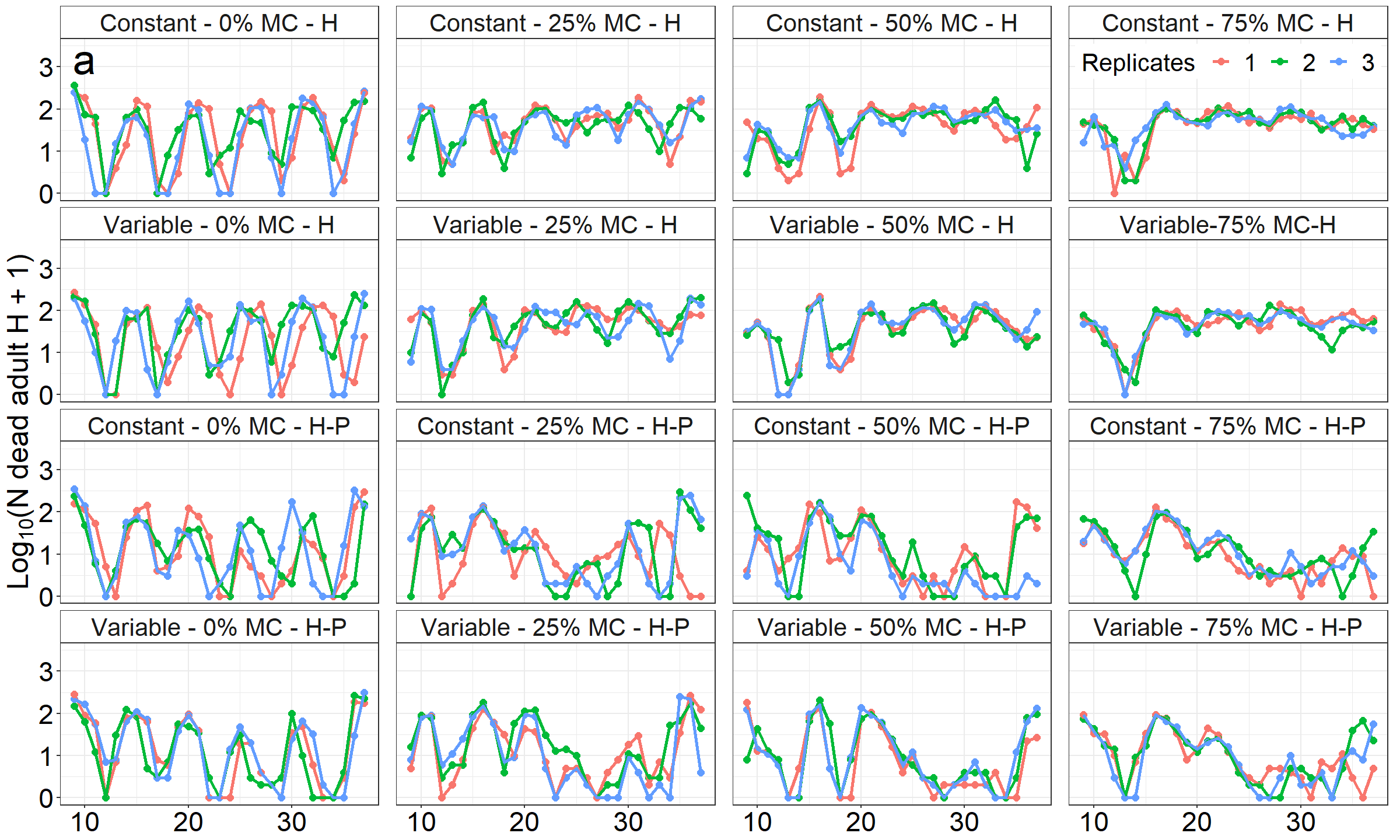


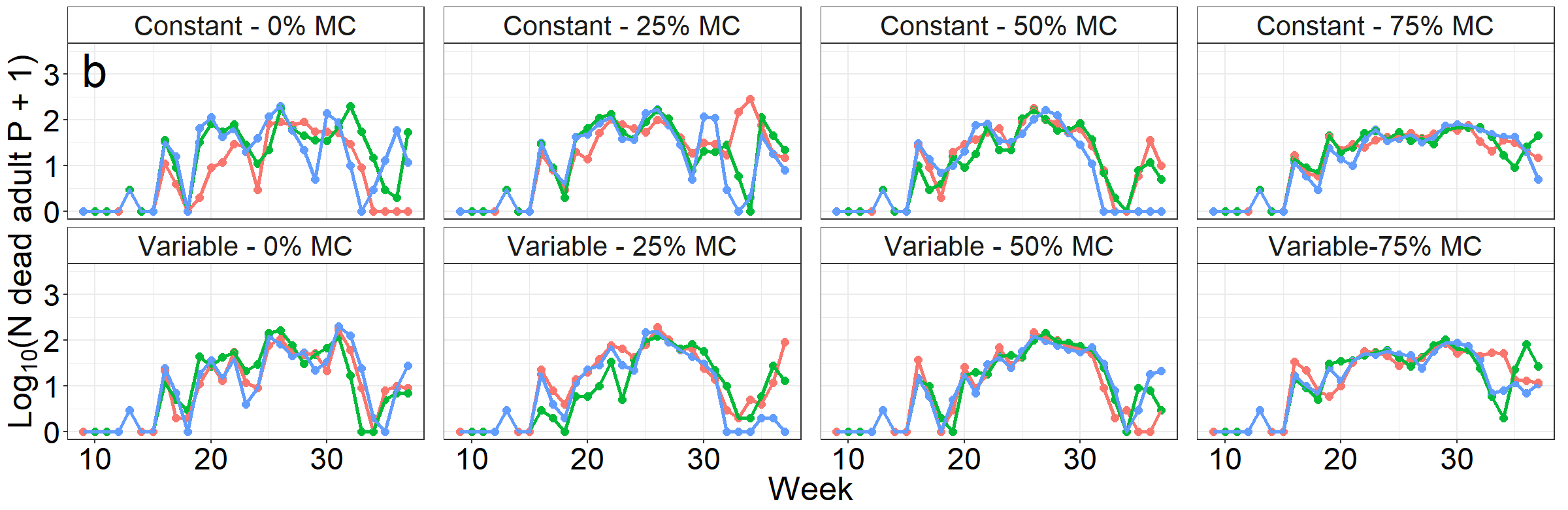


**Figure S1.** Raw time series of the number of dead adult (a) hosts and (b) parasitoids in experimental microcosms according to resource degradation (“0 % MC”, “25 % MC”, “50 % MC” and “75 % MC”), temperature (“Constant” *versus* “Variable”) and microcosm type (“host alone” (H) *versus* “host-parasitoid” (H-P)). “MC” refers to Methyl Cellulose, “H” refers to hosts and “P” refers to parasitoids. No adult parasitoid was found in the “Constant – 0 % MC” replicate 1, “Variable – 25 % MC” replicate 3 and “Constant – 50 % MC” replicate 3 on the last week of the experiment. An additional 4 weeks of monitoring of these replicates confirmed the extinction of the parasitoid population in “Variable – 25 % MC” replicate 3 and “Constant – 50 % MC”.

# Appendix S3 - AIC_c_ tables and tables of parameter estimates from the analysis of *Plodia* and *Venturia* life history traits

For each life history trait, an information-theoretic approach (Burnham & Anderson 2002) based on Akaike Information Criterion corrected for small sample size (AIC_c_) was used to compared the full model (as described in the main text) and all possible combinations of nested models using the *dredge* function of the *MuMIn* R package (Barton 2018). Supported models (ΔAIC_c_ ≤ 4) were then used to produce estimates of fixed effects and their 95 % confidence intervals. Averaging models with different contrasts (i.e., including different categorical variables, here: resource degradation, temperature variation and sex) yields meaningless estimates (Barton 2018). Therefore, parameter estimates of each fixed effect were obtained from the best supported model (i.e., with the smallest AIC_c_) including that fixed effect instead of using model averaging. Finally, whenever Bartlett’s tests revealed heterogeneity of variance between sexes or treatment groups, the best supported model was used to estimate variance parameters and their 95 % confidence intervals.

Table S1. AIC_c_ table from the analysis of unparasitised host juvenile stage duration.

The “+” and “-” symbols indicate the fixed effect(s) included in each model and the direction of the effect (i.e., positive or negative) on host juvenile stage duration. The model, number of parameters (*k*), AIC_c_ scores, model support (ΔAIC_c_) and model weights (*w_i_*) are provided for each model. “RD” refers to resource degradation and “Temp.” to temperature treatments. Only the best supported models (ΔAIC_c_ ≤ 4) are presented. Grey cells indicate the significance of parameter estimates in supported models based on 95 % confidence intervals.

| **Model** | **RD** | **RD^2^** | **Sex** | **Temp.** | **RD:Sex** | **RD:Temp.** | **RD^2^:Sex** | **RD^2^:Temp.** | **Sex:Temp.** | **RD:Sex:**  **Temp.** | **RD^2^:Sex:**  **Temp.** | ***k*** | **AICc** | Δ**AIC_c_** | ***w_i_*** |
| --- | --- | --- | --- | --- | --- | --- | --- | --- | --- | --- | --- | --- | --- | --- | --- |
| 432 | + | + | - | + |  | - |  | + | + |  |  | 10 | 1176.1 | 0.00 | 0.16 |
| 176 | + | + | - | + |  | - |  | + |  |  |  | 9 | 1176.5 | 0.40 | 0.13 |
| 448 | + | + | - | + | - | - |  | + | + |  |  | 11 | 1177.9 | 1.76 | 0.07 |
| 192 | + | + | - | + | - | - |  | + |  |  |  | 10 | 1178.0 | 1.85 | 0.06 |
| 496 | + | + | - | + |  | - | - | + | + |  |  | 11 | 1178.2 | 2.08 | 0.06 |
| 240 | + | + | - | + |  | - | - | + |  |  |  | 10 | 1178.4 | 2.30 | 0.05 |
| 400 | + | + | - | - |  |  |  | + | + |  |  | 9 | 1179.0 | 2.89 | 0.04 |
| 144 | + | + | - | - |  |  |  | + |  |  |  | 8 | 1179.4 | 3.26 | 0.03 |
| 512 | + | + | - | + | - | - | + | + | + |  |  | 12 | 1179.4 | 3.32 | 0.03 |
| 256 | + | + | - | + | - | - | + | + |  |  |  | 11 | 1179.5 | 3.41 | 0.03 |
| 272 | + | + | - | - |  |  |  |  | + |  |  | 8 | 1179.8 | 3.71 | 0.03 |
| 960 | + | + | - | + | - | - |  | + | + | + |  | 12 | 1180.1 | 4.00 | 0.02 |

Table S2. Parameter estimates from the analysis of unparasitised host juvenile stage duration.

Estimates of fixed effects and their 95% confidence intervals (CIs) are differences (in days) from the intercept (i.e., “constant temperature - female” group). Variance differed between temperature treatment groups (Bartlett’s test: K^2^ = 4.57, df = 1, P = 0.03). Estimates of the variance function and their 95% CIs compare to the variance of the intercept, here resource degradation treatment “0 % MC”, which is set to 1. Significant estimates (i.e., different from 0 for fixed effects and from 1 for the variance function) are in bold. “Model” indicates which supported model (Table S1) was used to obtain parameter estimates. “RD” refers to resource degradation and “Temp.” to temperature treatments.

| **Model** | **Fixed effects** | **Estimates** | **95% CIs** |
| --- | --- | --- | --- |
| **432** | **RD^†^** | 0.08 | **[0.003, 0.15]** |
| 432 | RD^2^ | 0.0009 | [-0.00002, 0.002] |
| 432 | Temp. |  |  |
|  | Variable | 0.36 | [-1.53, 2.24] |
| **432** | **Sex** |  |  |
|  | **Male** | **-1.83** | **[-3.07, -0.59]** |
| 448 | RD:Sex |  |  |
|  | Male | -0.01 | [-0.05, 0.0004] |
| 496 | RD^2^:Sex |  |  |
|  | Male | -0.0001 | [-0.0006, 0.004] |
| **432** | **RD:Temp.**^†^ |  |  |
|  | **Variable** | **-0.13** | **[-0.25, -0.02]** |
| **432** | **RD^2^:Temp.**^†^ |  |  |
|  | **Variable** | **0.002** | **[0.0006, 0.004]** |
| 432 | Sex:Temp. |  |  |
|  | Male:Variable | 1.62 | [-0.39, 3.64] |
| 960 | RD:Sex:Temp. |  |  |
|  | Male:Variable | 0.001 | [-0.08, 0.08] |
| **Model** | **Variance function** | **Estimates** | **95% CIs** |
| **432** | **Temp.** |  |  |
|  | **Variable** | **1.21** | **[1.01, 1.47]** |

^†^ Regression lines represented on Fig. 1a were obtained from the best supported model (432) without the explanatory variable ‘Sex’.

Table S3. AIC_c_ table from the analysis of parasitoid juvenile stage duration.

The “+” and “-” symbols indicate the fixed effect(s) included in each model and the direction of the effect (i.e., positive or negative) on parasitoid juvenile stage duration. The model, number of parameters (*k*), AIC_c_ scores, model support (ΔAIC_c_) and model weights (*w_i_*) are provided for each model. “RD” refers to resource degradation and “Temp.” to temperature treatments. Only the best supported models (ΔAIC_c_ ≤ 4) are presented. Grey cells indicate the significance of parameter estimates in supported models based on 95 % confidence intervals.

| **Model** | **RD** | **RD^2^** | **Temp.** | **RD:Temp.** | **RD^2^:Temp.** | ***k*** | **AICc** | Δ**AIC_c_** | ***w_i_*** |
| --- | --- | --- | --- | --- | --- | --- | --- | --- | --- |
| 24 | - | + | - |  | - | 6 | 901.6 | 0.00 | 0.30 |
| 32 | - | + | - | + | - | 7 | 901.7 | 0.11 | 0.28 |
| 8 | - | + | - |  |  | 5 | 901.8 | 0.26 | 0.26 |
| 16 | - | + | - | - |  | 6 | 902.8 | 1.23 | 0.16 |

Table S4. Parameter estimates from the analysis of parasitoid juvenile stage duration.

Estimates of fixed effects and their 95% confidence intervals (CIs) are differences (in days) from the intercept (i.e., “constant temperature” treatment). Significant estimates are in bold. “Model” indicates which supported model (Table S3) was used to obtain parameter estimates. “RD” refers to resource degradation and “Temp.” to temperature treatments.

| **Model** | **Fixed effects** | **Estimates** | **95% CIs** |
| --- | --- | --- | --- |
| 24 | RD | -0.010 | [-0.06, 0.04] |
| **24** | **RD^2^** | **0.001** | **[0.0006, 0.002]** |
| 24 | Temp. |  |  |
|  | Variable | -0.68^*^ | [-1.80, 0.44] |
| 32 | RD:Temp. |  |  |
|  | Variable | 0.07 | [-0.03, 0.17] |
| 24 | RD^2^:Temp. |  |  |
|  | Variable | -0.0003 | [-0.0006, 0.0001] |

^*^ Temperature variation shortened parasitoid juvenile stage duration (negative effect in all supported models). However, this negative effect was only significant in the supported model which did not include (non-significant) interaction terms between temperature treatment and resource degradation (Table S-C2.1). In this model, parasitoid juvenile stage duration was estimated to be 1.30 ± 0.78 days shorter in the variable than in the constant temperature treatment group.

Table S5. AIC_c_ table from the analysis of unparasitised host adult lifespan.

The “+” and “-” symbols indicate the fixed effect(s) included in each model and the direction of the effect (i.e., positive or negative) on host adult lifespan. The model, number of parameters (*k*), AIC_c_ scores, model support (ΔAIC_c_) and model weights (*w_i_*) are provided for each model. “RD” refers to resource degradation and “Temp.” to temperature treatments. Only the best supported models (ΔAIC_c_ ≤ 4) are presented. Grey cells indicate the significance of parameter estimates in supported models based on 95 % confidence intervals.

| **Model** | **RD** | **RD^2^** | **Sex** | **Temp.** | **RD:Sex** | **RD:Temp.** | **RD^2^:Sex** | **RD^2^:Temp.** | **Sex:Temp.** | **RD:Sex:**  **Temp.** | **RD^2^:Sex:**  **Temp.** | ***k*** | **AICc** | Δ**AIC_c_** | ***w_i_*** |
| --- | --- | --- | --- | --- | --- | --- | --- | --- | --- | --- | --- | --- | --- | --- | --- |
| 1 |  |  |  |  |  |  |  |  |  |  |  | 2 | 1010.6 | 0.00 | 0.19 |
| 2 | + |  |  |  |  |  |  |  |  |  |  | 3 | 1011.2 | 0.56 | 0.14 |
| 5 |  |  | - |  |  |  |  |  |  |  |  | 3 | 1012.6 | 1.98 | 0.07 |
| 9 |  |  |  | - |  |  |  |  |  |  |  | 3 | 1012.7 | 2.05 | 0.07 |
| 6 | + |  | - |  |  |  |  |  |  |  |  | 4 | 1012.9 | 2.31 | 0.06 |
| 4 | + | + |  |  |  |  |  |  |  |  |  | 4 | 1013.1 | 2.52 | 0.05 |
| 22 | - |  | - |  | + |  |  |  |  |  |  | 5 | 1013.1 | 2.53 | 0.05 |
| 10 | + |  |  | - |  |  |  |  |  |  |  | 4 | 1013.2 | 2.63 | 0.05 |

Table S6. Parameter estimates from the analysis of unparasitised host adult lifespan.

Estimates of fixed effects and their 95% confidence intervals (CIs) are differences (in days) from the intercept (i.e., “constant temperature - female” group). Significant estimates are in bold. “Model” indicates which supported model (Table S5) was used to obtain parameter estimates. “RD” refers to resource degradation and “Temp.” to temperature treatments.

| **Model** | **Fixed effects** | **Estimates** | **95% CIs** |
| --- | --- | --- | --- |
| 2 | RD | 0.008 | [-0.005, 0.02] |
| 4 | RD^2^ | 0.0001 | [-0.0004, 0.0006] |
| 9 | Temp. |  |  |
|  | Variable | -0.0300 | [-0.69, 0.63] |
| 5 | Sex |  |  |
|  | Male | -0.09 | [-0.76, 0.57] |
| 22 | RD:Sex |  |  |
|  | Male | 0.02 | [-0.008, 0.04] |

Table S7. AIC_c_ table from the analysis of parasitoid adult lifespan.

The “+” and “-” symbols indicate the fixed effect(s) included in each model and the direction of the effect (i.e., positive or negative) on parasitoid adult lifespan. The model, number of parameters (*k*), AIC_c_ scores, model support (ΔAIC_c_) and model weights (*w_i_*) are provided for each model. “RD” refers to resource degradation and “Temp.” to temperature treatments. Only the best supported models (ΔAIC_c_ ≤ 4) are presented. Grey cells indicate the significance of parameter estimates in supported models based on 95 % confidence intervals.

| **Model** | **RD** | **RD^2^** | **Temp.** | **RD:Temp.** | **RD^2^:Temp.** | ***k*** | **AICc** | Δ**AIC_c_** | ***w_i_*** |
| --- | --- | --- | --- | --- | --- | --- | --- | --- | --- |
| 32 | - | + | - | + | - | 7 | 319.9 | 0 | 0.77 |

Table S8. Parameter estimates from the analysis of parasitoid adult lifespan.

Estimates of fixed effects and their 95% confidence intervals (CIs) are differences (in days) from the intercept (i.e., “constant temperature” treatment). Significant estimates are in bold. “Model” indicates which supported model (Table S7) was used to obtain parameter estimates. “RD” refers to resource degradation and “Temp.” to temperature treatments.

| **Model** | **Fixed effects** | **Estimates** | **95% CIs** |
| --- | --- | --- | --- |
| 32 | RD | -0.01 | [-0.03, 0.005] |
| **32** | **RD^2^** | **0.0002** | **[0.00006, 0.0004]** |
| 32 | Temp. |  |  |
|  | Variable | -0.07 | [-0.43, 0.29] |
| **32** | **RD:Temp.** |  |  |
|  | **Variable** | **0.03** | **[0.01, 0.06]** |
| **32** | **RD^2^:Temp.** |  |  |
|  | **Variable** | **-0.0005** | **[-0.0007, -0.0002]** |

Table S9. AIC_c_ table from the analysis of unparasitised host adult mid-femur length.

The “+” and “-” symbols indicate the fixed effect(s) included in each model and the direction of the effect (i.e., positive or negative) on host adult femur length. The model, number of parameters (*k*), AIC_c_ scores, model support (ΔAIC_c_) and model weights (*w_i_*) are provided for each model. “RD” refers to resource degradation and “Temp.” to temperature treatments. Only the best supported models (ΔAIC_c_ ≤ 4) are presented. Grey cells indicate the significance of parameter estimates in supported models based on 95 % confidence intervals.

| **Model** | **RD** | **RD^2^** | **Sex** | **Temp.** | **RD:Sex** | **RD:Temp.** | **RD^2^:Sex** | **RD^2^:Temp.** | **Sex:Temp.** | **RD:Sex:**  **Temp.** | **RD^2^:Sex:**  **Temp.** | ***k*** | **AICc** | Δ**AIC_c_** | ***w_i_*** |
| --- | --- | --- | --- | --- | --- | --- | --- | --- | --- | --- | --- | --- | --- | --- | --- |
| 6 | - |  | - |  |  |  |  |  |  |  |  | 4 | -348.3 | 0.00 | 0.22 |
| 8 | - | + | - |  |  |  |  |  |  |  |  | 5 | -346.9 | 1.45 | 0.10 |
| 22 | - |  | - |  | + |  |  |  |  |  |  | 5 | -346.8 | 1.53 | 0.10 |
| 14 | - |  | - | - |  |  |  |  |  |  |  | 5 | -346.2 | 2.07 | 0.08 |
| 72 | - | + | - |  |  |  | + |  |  |  |  | 6 | -345.3 | 3.04 | 0.05 |
| 24 | - | + | - |  | + |  |  |  |  |  |  | 6 | -345.2 | 3.16 | 0.04 |
| 46 | - |  | - | - |  | + |  |  |  |  |  | 6 | -344.9 | 3.44 | 0.04 |
| 16 | - | + | - | - |  |  |  |  |  |  |  | 6 | -344.8 | 3.54 | 0.04 |
| 30 | - |  | - | - | + |  |  |  |  |  |  | 6 | -344.7 | 3.62 | 0.04 |
| 270 | - |  | - | + |  |  |  |  | - |  |  | 6 | -344.4 | 3.89 | 0.03 |

Table S10. Parameter estimates from the analysis of unparasitised host adult mid-femur length.

Estimates of fixed effects and their 95% confidence intervals (CIs) are differences (in days) from the intercept (i.e., “constant temperature - female” group). Significant estimates are in bold. “Model” indicates which supported model (Table S9) was used to obtain parameter estimates. “RD” refers to resource degradation and “Temp.” to temperature treatments.

| **Model** | **Fixed effects** | **Estimates** | **95% CIs** |
| --- | --- | --- | --- |
| **6** | **RD^†^** | **-0.002** | **[-0.002, -0.001]** |
| 8 | RD^2^ | 0.00001 | [-0.000014, 0.00003] |
| 14 | Temp. |  |  |
|  | Variable | -0.002 | [-0.03, 0.03] |
| **6** | **Sex** |  |  |
|  | **Male** | **-0.18** | **[-0.21, -0.15]** |
| 22 | RD:Sex |  |  |
|  | Male | 0.0004 | [-0.007, 0.002] |
| 72 | RD^2^:Sex |  |  |
|  | Male | 0.00006 | [-0.00001, 0.00002] |
| 46 | RD:Temp. |  |  |
|  | Variable | 0.0005 | [-0.006, 0.002] |
| 270 | Sex:Temp. |  |  |
|  | Male:Variable | -0.02 | [-0.07, 0.04] |

^†^ The regression line represented on Fig. 2a in the main text was obtained from the best supported model (6) without the explanatory variable ‘Sex’.

Table S11. AIC_c_ table from the analysis of parasitoid adult hind tibia length.

The “+” and “-” symbols indicate the fixed effect(s) included in each model and the direction of the effect (i.e., positive or negative) on parasitoid adult hind tibia length. The model, number of parameters (*k*), AIC_c_ scores, model support (ΔAIC_c_) and model weights (*w_i_*) are provided for each model. “RD” refers to resource degradation and “Temp.” to temperature treatments. Only the best supported models (ΔAIC_c_ ≤ 4) are presented. Grey cells indicate the significance of parameter estimates in supported models based on 95 % confidence intervals.

| **Model** | **RD** | **RD^2^** | **Temp.** | **RD:Temp.** | **RD^2^:Temp.** | ***k*** | **AICc** | Δ**AIC_c_** | ***w_i_*** |
| --- | --- | --- | --- | --- | --- | --- | --- | --- | --- |
| 4 | + | - |  |  |  | 4 | -312.3 | 0.00 | 0.29 |
| 2 | - |  |  |  |  | 3 | -312 | 0.30 | 0.25 |
| 8 | + | - | - |  |  | 5 | -310.7 | 1.54 | 0.14 |
| 6 | - |  | - |  |  | 4 | -310.4 | 1.83 | 0.12 |
| 24 | + | - | + |  | - | 6 | -309.3 | 2.94 | 0.07 |
| 16 | + | - | + | - |  | 6 | -309 | 3.26 | 0.06 |
| 14 | - |  | + | - |  | 5 | -308.7 | 3.56 | 0.05 |

Table S12. Parameter estimates from the analysis of parasitoid adult hind tibia length.

Estimates of fixed effects and their 95% confidence intervals (CIs) are differences (in days) from the intercept (i.e., “constant temperature” treatment). Significant estimates are in bold. “Model” indicates which supported model (Table S11) was used to obtain parameter estimates. “RD” refers to resource degradation and “Temp.” to temperature treatments.

| **Model** | **Fixed effects** | **Estimates** | **95% CIs** |
| --- | --- | --- | --- |
| 4 | RD | 0.0002^*^ | [-0.002, 0.002] |
| 4 | RD^2^ | -0.00002 | [-0.00004, 0.000005] |
| 8 | Temp. |  |  |
|  | Variable | -0.01 | [-0.04, 0.02] |
| 16 | RD:Temp. |  |  |
|  | Variable | -0.0004 | [-0.0001, 0.0007] |
| 24 | RD^2^:Temp. |  |  |
|  | Variable | -0.00001 | [-0.00002, 0.000008] |

^*^ Resource degradation as a linear term had a significant negative effect on parasitoid adult hind tibia length in all the supported models which did not include resource degradation as a quadratic term (Table S11, estimate from model 2 = -0.001 ± 0.0005).

Table S13. AIC_c_ table from the analysis of unparasitised host egg viability.

The “+” and “-” symbols indicate the fixed effect(s) included in each model and the direction of the effect (i.e., positive or negative) on host egg viability. The model, number of parameters (*k*), AIC_c_ scores, model support (ΔAIC_c_) and model weights (*w_i_*) are provided for each model. “RD” refers to resource degradation and “Temp.” to temperature treatments. Only the best supported models (ΔAIC_c_ ≤ 4) are presented. Grey cells indicate the significance of parameter estimates in supported models based on 95 % confidence intervals.

| **Model** | **RD** | **RD^2^** | **Temp.** | **RD:Temp.** | **RD^2^:Temp.** | ***k*** | **AICc** | Δ**AIC_c_** | ***w_i_*** |
| --- | --- | --- | --- | --- | --- | --- | --- | --- | --- |
| 5 |  |  | - |  |  | 2 | 186.6 | 0.00 | 0.28 |
| 1 |  |  |  |  |  | 1 | 186.7 | 0.03 | 0.28 |
| 6 | + |  | - |  |  | 3 | 188.6 | 1.98 | 0.10 |
| 2 | + |  |  |  |  | 2 | 188.6 | 2.00 | 0.10 |
| 14 | - |  | - | + |  | 4 | 188.9 | 2.25 | 0.09 |

Table S14. Parameter estimates from the analysis of unparasitised host host egg viability.

Estimates of fixed effects and their 95% confidence intervals (CIs) are differences (in days) from the intercept (i.e., “constant temperature” treatment). Significant estimates are in bold. “Model” indicates which supported model (Table S13) was used to obtain parameter estimates. “RD” refers to resource degradation and “Temp.” to temperature treatments.

| **Model** | **Fixed effects** | **Estimates** | **95% CIs** |
| --- | --- | --- | --- |
| 6 | RD | 0.002 | [-0.01, 0.02] |
| 5 | Temp. |  |  |
|  | Variable | -0.59 | [-1.43, 0.21] |
| 5 | RD:Temp. |  |  |
|  | Variable | 0.02 | [-0.009, 0.05] |

# Appendix S4 – Cyclical behaviour of host time series

The cyclical behaviour of host time series was investigated using the *periodogram* procedure of the *TSA* R package. In spectral analysis, which investigates the frequency properties of time series, periodograms are used to identify the dominant frequencies in time series, which are indicated by the highest peaks in the periodogram (Cryer & Chan 2009).

In “host alone” (H) microcosms, a dominant frequency of 0.16667 and/or 0.2 was detected in the host dynamics of all but one microcosm (PiCst0-r2) under 0, 25 and 50 % MC. However, the intensity of the signal decreased with resource degradation and no dominant frequency was detected in microcosms under 75% MC (Figure S1a). A dominant frequency of 0.1667-0.2 indicate the occurrence of host population cycles with a period of 5-6 weeks, which is consistent with previous studies of this system at constant temperature and high host quality resource (Sait, Begon & Thompson 1994; Begon, Sait & Thompson 1996; Briggs *et al.* 2000). This matches the generation time of about 35-42 days we observed in the life history assay (see Figure 1a in the main text; the average generation time in each resource degradation treatment can be obtained by adding 4 days, the average egg stage duration in all treatments (results not shown), to juvenile stage duration). The decrease in the intensity of the dominant frequency signal seen in periodograms (Figure S1a) indicates a dampening of these generation cycles with resource degradation (see Figure 3a in the main text). In “host-parasitoid” microcosms (H-P), a weak dominant frequency in host dynamics was only detected in two microcosms under 25 % MC (Figure S1b). This indicates that the presence of parasitoids altered the cyclic behaviour of host dynamics under all levels of resource degradation.

**
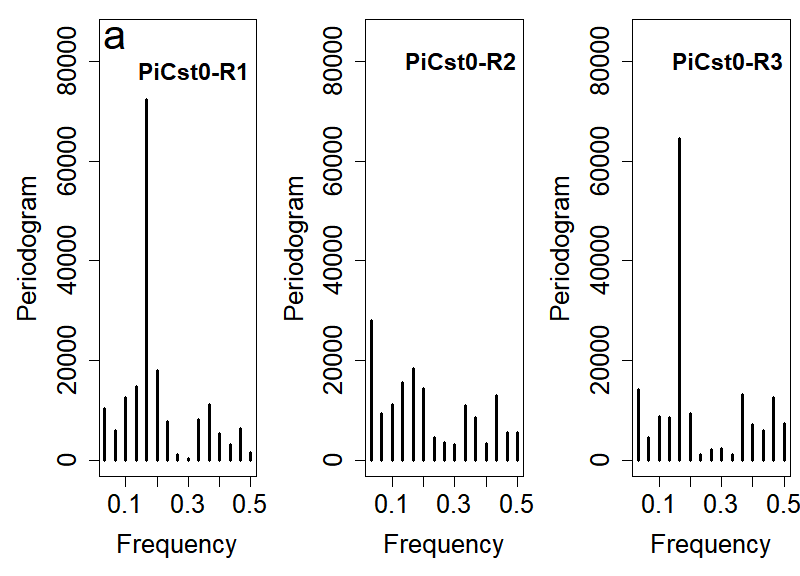
**
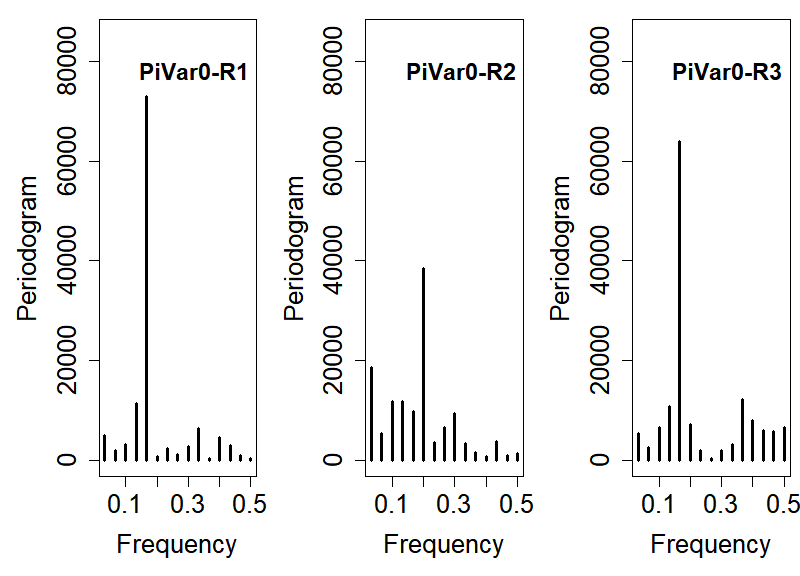


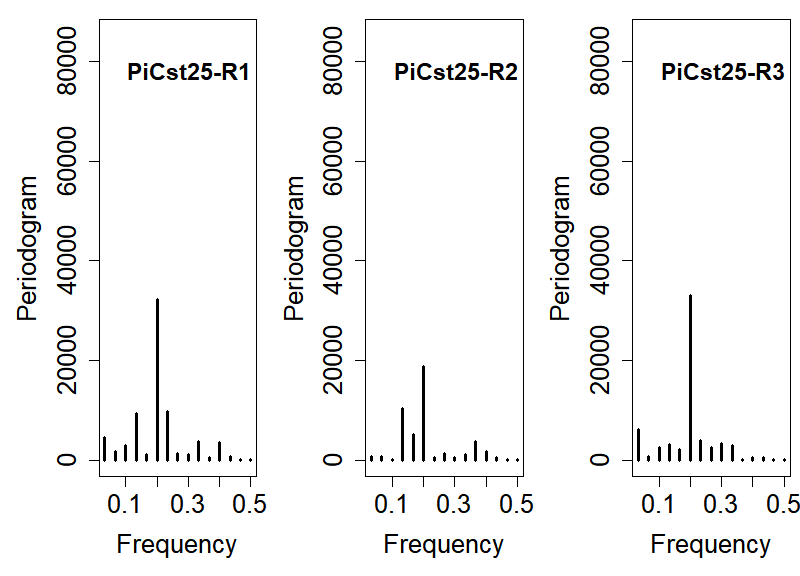

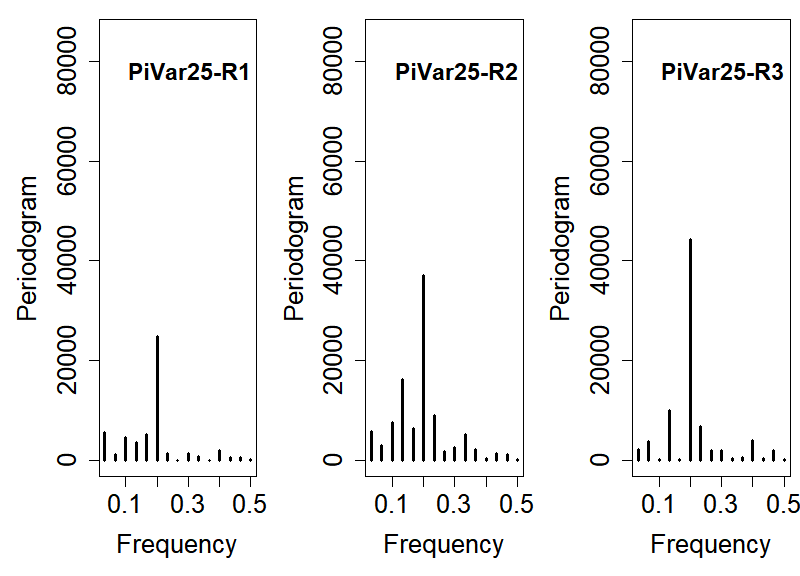


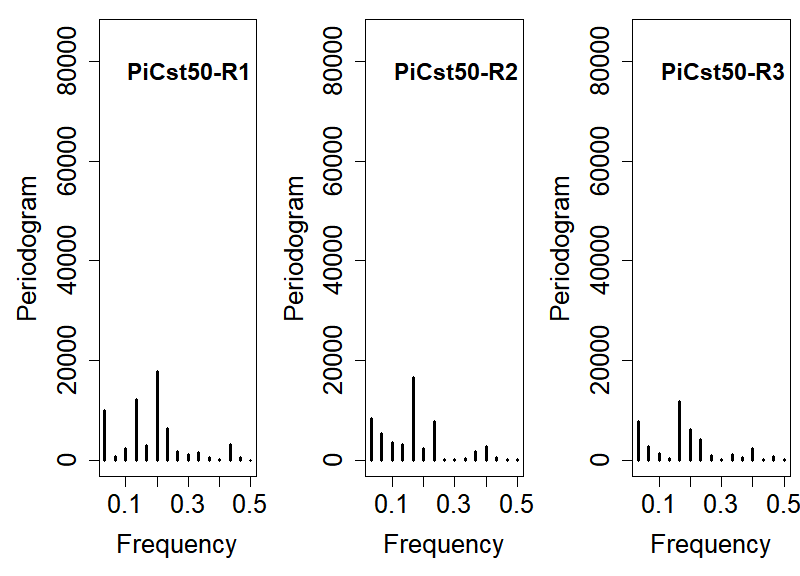
7
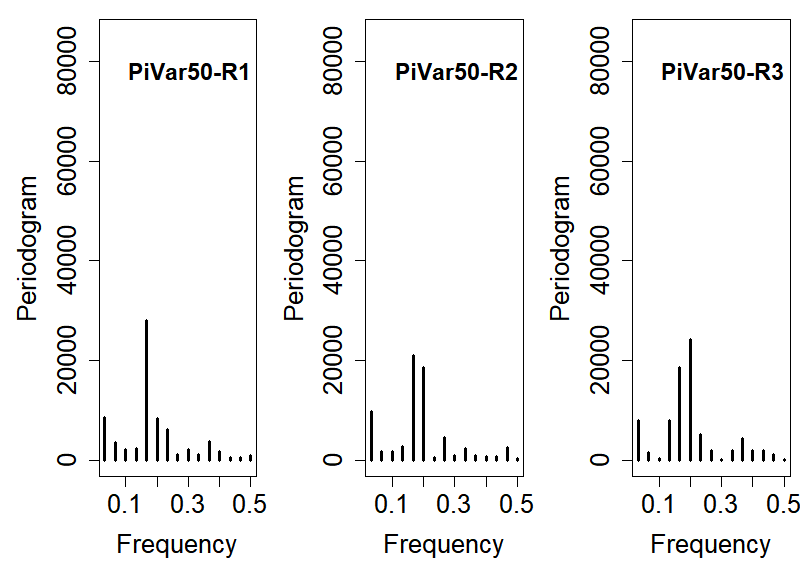


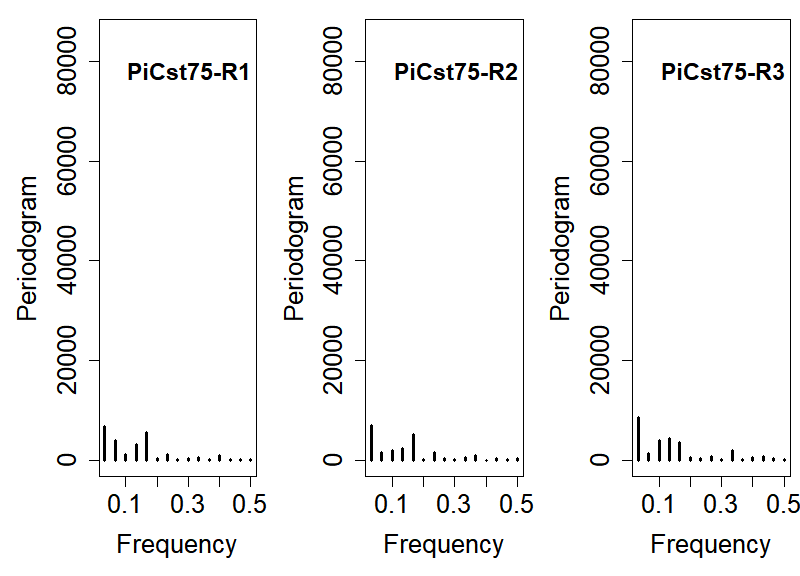

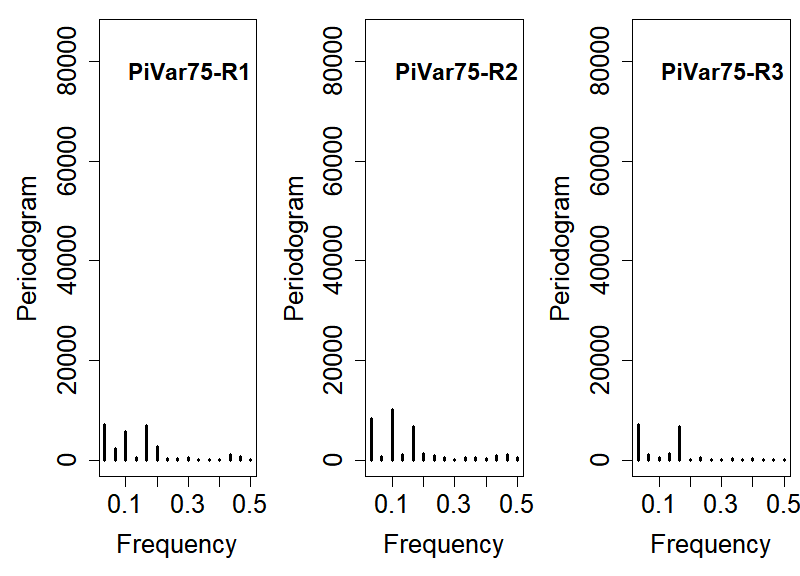


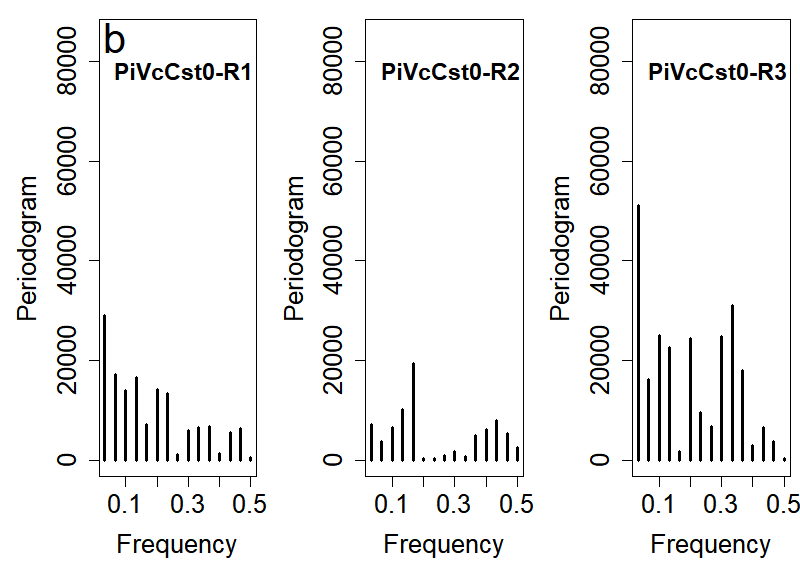

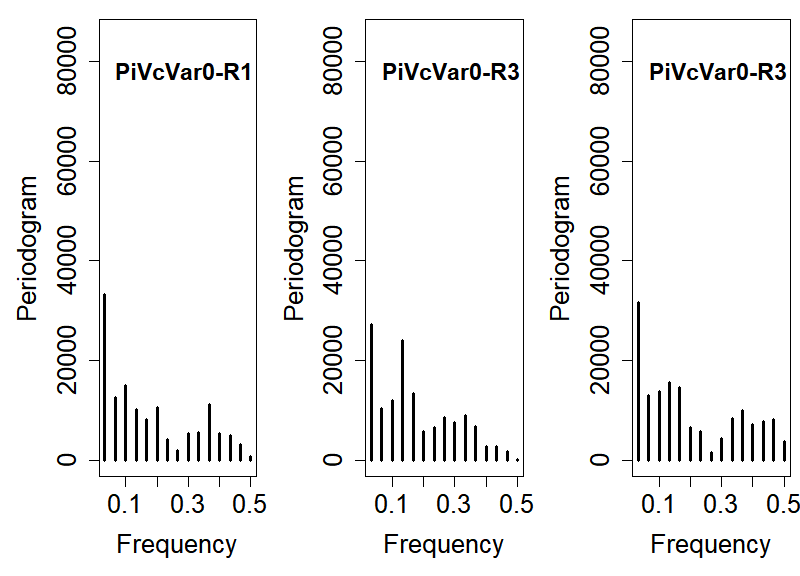


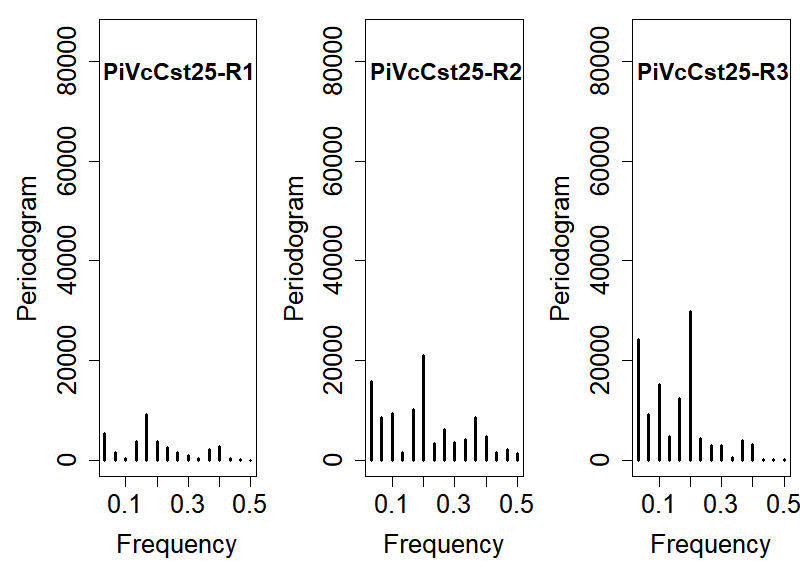

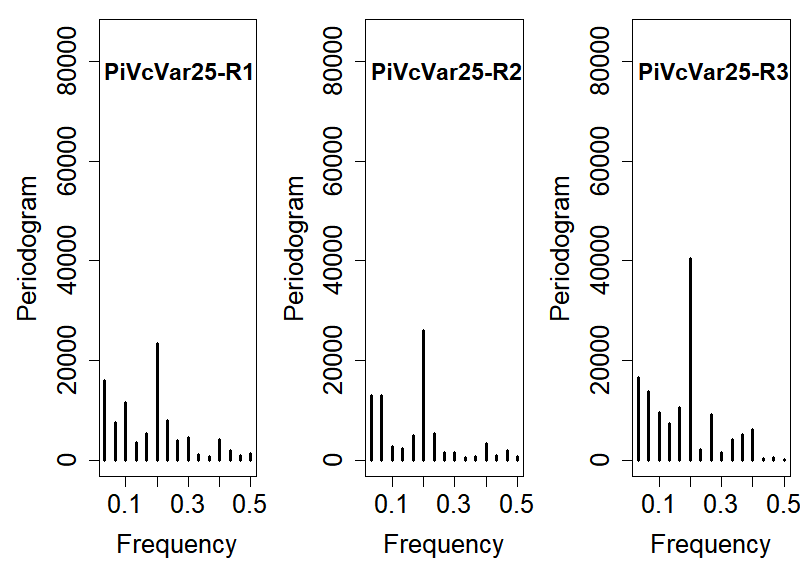


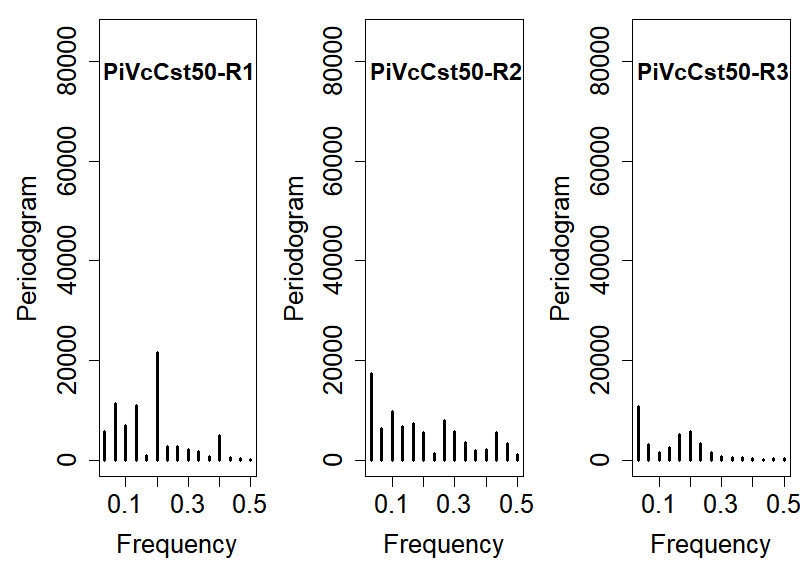

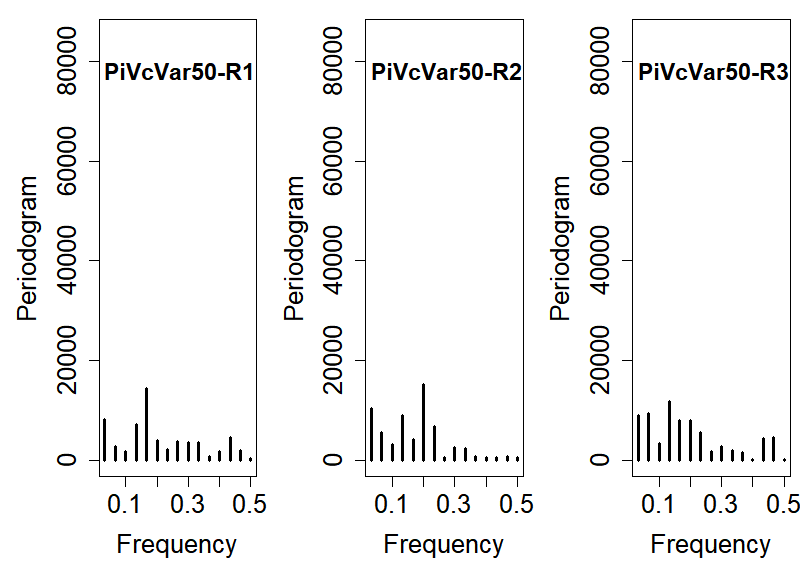


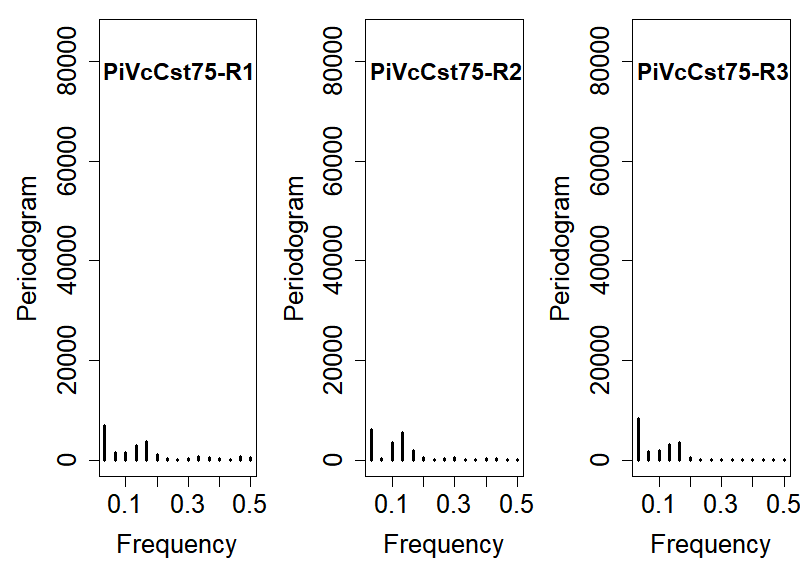

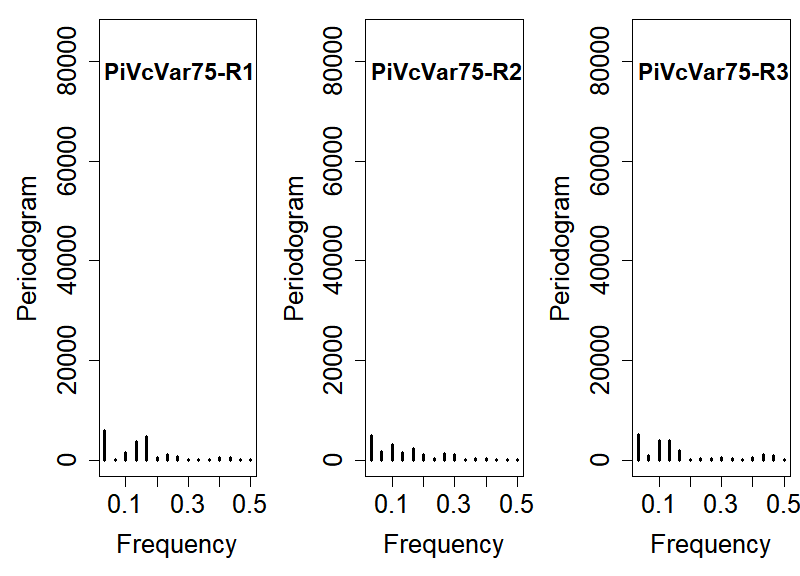


**Figure S1.** Periodograms for each of the 24 host time series in (a) host alone microcosms and in (b) host-parasitoid microcosms. Fourier frequencies are represented on the x-axis and spectrum estimates at given frequencies are represented on the y-axis (Cryer & Chan 2009). The highest peaks in the periodograms indicate the dominant frequencies or cycle periods (with *T* = 1/*f*, where *T* is the period (or cycle length) and *f* the frequency). The microcosm code at the top of each panel refers to microcosm type (“Pi” for host alone and “PiVc” for host-parasitoid), temperature treatments (“Cst” for “Constant” and “Var” for “Variable”), resource degradation treatments (0, 25, 50 and 75 % MC) and replicates (R1-3).

# Appendix S5 – AIC_c_ tables and tables of parameter estimates from the analysis of the change in normalized amplitudes of fluctuations of adult host abundance through time

The change in amplitude of weekly fluctuations of normalized adult host time series were analyzed following Fussmann *et al.* (2014). Normalized time series were obtained by dividing the predictions of GAMs by the predictions of generalized linear models with a linear regression for each time series. The amplitude of the first intrinsic mode function (IMF) of the normalized time series and time steps (in weeks) at which they occurred were extracted using the *emd* procedure of the *EMD* R package (Kim & Oh 2009). An Empirical Mode Decomposition decomposes a signal into a series of IMFs which characterize the intrinsic oscillations of the components of a signal and are defined by the signals’ local extrema (i.e., minima and maxima) and zero-crossings. An oscillating wave is considered to be an IMF if the numbers of extrema and zero-crossings differ only by one and if the local average is zero. If these two conditions are not satisfied after extracting the first IMF, a second IMF will be extracted from the residual signal and so on (Kim & Oh 2009). Here, we did not analyze the amplitude of the second IMFs as none were detected in 18 out of 48 host populations. Parasitoid time series were too short to allow amplitudes analyses (see Figure S1b in Appendix S2).

Table S1. AIC_c_ table from the analysis of the temporal change of adult host normalized amplitudes.

The “×” symbols indicate the fixed effects included in the model. The number of parameters (*k*), AIC_c_ scores, model support (ΔAIC_c_) and model weights (*w_i_*) are provided for the model. Only the best supported models (ΔAIC_c_ ≤ 4) is presented. Grey cells indicate the significance of parameter estimates based on 95 % confidence intervals.

| **Model** | **Week** | **Week^2^** | **Treatment** | **Treatment:week** | **Treatment:week^2^** | ***k*** | **AIC_c_** | Δ**AIC_c_** | ***w_i_*** |
| --- | --- | --- | --- | --- | --- | --- | --- | --- | --- |
| 32 | × | × | × | × | × | 66 | 136.3 | 0 | 1 |

Table S2. Estimates of fixed effects from the best supported model of adult host normalized amplitudes.

Estimates of fixed effects and their 95% confidence intervals (CIs) are differences (in normalized amplitude) from the intercept (i.e., “Constant - 0 % MC - H” treatment group). Significant estimates are in bold. “Constant” and “Variable” refer to the temperature treatments, “MC” refers to Methyl Cellulose and “H” and “H-P” refer to “host alone” and “host-parasitoid” microcosms.

| **Fixed effects** | **Estimates** | **95% CIs** |
| --- | --- | --- |
| **Week** | **-0.20** | **[-0.30, -0.10]** |
| **Week^2^** | **0.007** | **[0.004, 0.01]** |
| **Treatment** |  |  |
| **Constant - 25 % MC - H** | **-1.36** | **[-2.11, -0.60]** |
| **Constant - 50 % MC - H** | **-0.98** | **[-1.85, -0.10]** |
| **Constant - 75 % MC - H** | **-1.94** | **[-2.67, -1.21]** |
| **Variable - 0 % MC - H** | **1.37** | **[0.12, 2.61]** |
| **Variable - 25 % MC - H** | **-1.04** | **[-1.80, -0.28]** |
| **Variable - 50 % MC - H** | **-1.65** | **[-2.41, -0.88]** |
| **Variable - 75 % MC - H** | **-1.94** | **[-2.69, -1.18]** |
| Constant - 0 % MC - H-P | 0.32 | [-0.65, 1.30] |
| Constant - 25 % MC - H-P | 0.52 | [-0.38, 1.42] |
| Constant - 50 % MC - H-P | 0.38 | [-0.85, 1.62] |
| **Constant - 75 % MC - H-P** | **-1.39** | **[-2.14, -0.65]** |
| Variable - 0 % MC - H-P | 0.42 | [-0.51, 1.35] |
| Variable - 25 % MC - H-P | -0.06 | [-1.18, 1.06] |
| Variable - 50 % MC - H-P | -1.13 | [-2.44, 0.18] |
| **Variable - 75 % MC - H-P** | **-1.29** | **[-2.09, -0.48]** |
| **Treatment:week** |  |  |
| **Constant - 25 % MC - H:week** | **0.12** | **[0.01, 0.23]** |
| Constant - 50 % MC - H:week | 0.09 | [-0.03, 0.22] |
| **Constant - 75 % MC - H:week** | **0.15** | **[0.05, 0.26]** |
| Variable - 0 % MC - H:week | -0.16 | [-0.34, 0.02] |
| Variable - 25 % MC - H:week | 0.09 | [-0.02, 0.20] |
| **Variable - 50 % MC - H:week** | **0.23** | **[0.12, 0.34]** |
| **Variable - 75 % MC - H:week** | **0.15** | **[0.04, 0.26]** |
| Constant - 0 % MC - H-P:week | -0.10 | [-0.24, 0.04] |

Table S2 to be continued on the next page.

| **Fixed effects** | **Estimates** | **95% CIs** |
| --- | --- | --- |
| Constant - 25 % MC - H-P:week | -0.13 | [-0.26, 0.01] |
| Constant - 50 % MC - H-P:week | 0.01 | [-0.18, 0.19] |
| **Constant - 75 % MC - H-P:week** | **0.15** | **[0.04, 0.26]** |
| Variable - 0 % MC - H-P:week | -0.07 | [-0.20, 0.07] |
| Variable - 25 % MC - H-P:week | 0.02 | [-0.15, 0.19] |
| **Variable - 50 % MC - H-P:week** | **0.29** | **[0.09, 0.49]** |
| **Variable - 75 % MC - H-P:week** | **0.12** | **[0.01, 0.24]** |
| **Treatment:week^2^** |  |  |
| **Constant - 25 % MC - H:week^2^** | **-0.005** | **[-0.008, -0.001]** |
| **Constant - 50 % MC - H:week^2^** | **-0.004** | **[-0.008, -0.00004]** |
| **Constant - 75 % MC - H:week^2^** | **-0.006** | **[-0.009, -0.002]** |
| Variable - 0 % MC - H:week^2^ | 0.005 | [-0.001, 0.01] |
| Variable - 25 % MC - H:week^2^ | -0.003 | [-0.007, 0.00007] |
| **Variable - 50 % MC - H:week^2^** | **-0.009** | **[-0.012, -0.005]** |
| **Variable - 75 % MC - H:week^2^** | **-0.005** | **[-0.009, -0.002]** |
| Constant - 0 % MC - H-P:week^2^ | 0.003 | [-0.001, 0.008] |
| Constant - 25 % MC - H-P:week^2^ | 0.004 | [-0.0004, 0.008] |
| Constant - 50 % MC - H-P:week^2^ | -0.003 | [-0.009, 0.003] |
| **Constant - 75 % MC - H-P:week^2^** | **-0.006** | **[-0.009, -0.002]** |
| Variable - 0 % MC - H-P:week^2^ | 0.002 | [-0.003, 0.006] |
| Variable - 25 % MC - H-P:week^2^ | -0.001 | [-0.007, 0.004] |
| **Variable - 50 % MC - H-P:week^2^** | **-0.011** | **[-0.018, -0.004]** |
| **Variable - 75 % MC - H-P:week^2^** | **-0.005** | **[-0.009, -0.001]** |

Table S3. Estimates of the variance function, random effect and correlation structure of the best supported model of adult host normalized amplitudes.

Estimates of the variance function and their 95% confidence intervals (CIs) compare to the variance of the intercept, here the treatment group “Constant - 0 % MC - H”, which is set to 1. Significant estimates (i.e., different from 1 for the variance function and from 0 for the random effect and correlation structure) are in bold. “Constant” and “Variable” refer to the temperature treatments, “MC” refers to Methyl Cellulose and “H” and “H-P” refer to “host alone” and “host-parasitoid” microcosms.

| **Variance function** | **Estimates** | **95% CIs** |
| --- | --- | --- |
| **Treatment** |  |  |
| **Constant - 25 % MC - H** | **0.45** | **[0.30, 0.68]** |
| Constant - 50 % MC - H | 0.85 | [0.57, 1.27] |
| **Constant - 75 % MC - H** | **0.21** | **[0.14, 0.33]** |
| **Variable - 0 % MC - H** | **1.50** | **[1.01, 2.23]** |
| **Variable - 25 % MC - H** | **0.55** | **[0.37, 0.82]** |
| Variable - 50 % MC - H | 0.79 | [0.53, 1.18] |
| **Variable - 75 % MC - H** | **0.33** | **[0.21, 0.50]** |
| Constant - 0 % MC - H-P | 0.92 | [0.62, 1.36] |
| Constant - 25 % MC - H-P | 1.00 | [0.65, 1.55] |
| **Constant - 50 % MC - H-P** | **1.61** | **[1.09, 2.38]** |
| **Constant - 75 % MC - H-P** | **0.56** | **[0.37, 0.84]** |
| Variable - 0 % MC - H-P | 0.83 | [0.57, 1.22] |
| **Variable - 25 % MC - H-P** | **1.49** | **[1.01, 2.19]** |
| **Variable - 50 % MC - H-P** | **2.04** | **[1.34, 3.10]** |
| **Variable - 75 % MC - H-P** | **0.50** | **[0.33, 0.75]** |
| **Random effect** | **Estimate** | **95% CIs** |
| **Microcosm identity** | **0.07** | **[0.03, 0.16]** |
| **Correlation structure** | **Estimate** | **95% CIs** |
| **corAR1( )** | **Phi = 0.32** | **[0.45, 0.56]** |

# Appendix S6 – Mean and variability of the abundance of dead adult hosts and parasitoids

The overall mean and the variability of the abundance of dead adult hosts and parasitoid was analysed using an information-theoretic approach (Burnham & Anderson 2002) based on Akaike Information Criterion corrected for small sample size (AIC_c_). The support of the full models (as described in the main text) and all possible combinations of nested models was compared using the *dredge* function of the *MuMIn* R package (Barton 2018). Supported models (ΔAIC_c_ ≤ 4) were then used to produce estimates of fixed effects and their 95 % confidence intervals. Averaging models with different contrasts (i.e., including different categorical variables, here: resource degradation, temperature variation and sex) yields meaningless estimates (Barton 2018). Therefore, parameter estimates of each fixed effect were obtained from the best supported model (i.e., with the smallest AIC_c_) including that fixed effect instead of using model averaging. Finally, whenever Bartlett’s tests revealed heterogeneity of variance between treatment groups, the best supported model was used to estimate variance parameters and their 95 % confidence intervals.

Table S1. AIC_c_ table from the analysis of the mean abundance of dead adult hosts in experimental microcosms.

The “+” and “-” symbols indicate the fixed effect(s) included in each model and the direction of the effect (i.e., positive or negative) on the mean abundance of dead adult hosts. The model, number of parameters (*k*), AIC_c_ scores, model support (ΔAIC_c_) and model weights (*w_i_*) are provided for each model. “RD” refers to resource degradation, “MT” to microcosm type and “Temp.” to temperature treatments. Only the best supported models (ΔAIC_c_ ≤ 4) are presented. Grey cells indicate the significance of parameter estimates in supported models based on 95 % confidence intervals.

| **Model** | **RD** | **RD^2^** | **MT** | **Temp.** | **RD:MT** | **RD:Temp.** | **RD^2^:MT** | **RD^2^:Temp.** | **MT:Temp.** | **RD:MT:**  **Temp** | **RD^2^:MT:**  **Temp** | **k** | **AICc** | **ΔAICc** | ***w_i_*** |
| --- | --- | --- | --- | --- | --- | --- | --- | --- | --- | --- | --- | --- | --- | --- | --- |
| 22 | - |  | - |  | - |  |  |  |  |  |  | 5 | 303.6 | 0.00 | 0.227 |
| 30 | - |  | - | + | - |  |  |  |  |  |  | 6 | 304.1 | 0.46 | 0.181 |
| 24 | - | - | - |  | - |  |  |  |  |  |  | 6 | 304.8 | 1.21 | 0.124 |
| 32 | - | - | - | + | - |  |  |  |  |  |  | 7 | 305.3 | 1.73 | 0.096 |
| 62 | - |  | - | + | - | + |  |  |  |  |  | 7 | 306.8 | 3.20 | 0.046 |
| 286 | - |  | - | + | - |  |  |  | - |  |  | 7 | 306.8 | 3.20 | 0.046 |
| 88 | + | - | - |  | - |  | + |  |  |  |  | 7 | 306.9 | 3.26 | 0.044 |
| 96 | + | - | - | + | - |  | + |  |  |  |  | 8 | 307.5 | 3.89 | 0.033 |

Table S2. Parameter estimates from the analysis of the mean abundance of dead adult hosts in experimental microcosms.

Estimates of fixed effects and their 95% confidence intervals (CIs) are differences (in number of dead adult hosts) from the intercept (i.e., “constant temperature - H microcosms” treatment group). Significant estimates are in bold. “Model” indicates which supported model (Table S1) was used to obtain parameter estimates. “RD” refers to resource degradation, “MT” to microcosm type, “H-P” to the host-parasitoid microcosms and “Temp.” to temperature treatments.

| **Model** | **Fixed effects** | **Estimates** | **95% CIs** |
| --- | --- | --- | --- |
| **22** | **RD** | **-0.12** | **[-0.20, -0.04]** |
| 24 | RD^2^ | -0.001 | [-0.004, 0.001] |
| **22** | **MT** |  |  |
|  | **H-P** | **-18.61** | **[-23.77, -13.45]** |
| 30 | Temp. |  |  |
|  | Variable | 2.13 | [-0.92, 5.18] |
| **22** | **RD:MT** |  |  |
|  | **H-P** | **-0.21** | **[-0.32, -0.10]** |
| 62 | RD:Temp. |  |  |
|  | Variable | 0.003 | [-0.11, 0.11] |
| 88 | RD^2^:MT |  |  |
|  | H-P | 0.002 | [-0.003, 0.007] |
| 286 | MT:Temp. |  |  |
|  | H-P:Variable | -0.14 | [-6.32, 6.04] |

Table S3. AIC_c_ table from the analysis of variability of the abundance of dead adult hosts in experimental microcosms.

The “+” and “-” symbols indicate the fixed effect(s) included in each model and the direction of the effect (i.e., positive or negative) on the variability of the abundance of dead adult hosts. The model, number of parameters (*k*), AIC_c_ scores, model support (ΔAIC_c_) and model weights (*w_i_*) are provided for each model. “RD” refers to resource degradation, “MT” to microcosm type and “Temp.” to temperature treatments. Only the best supported models (ΔAIC_c_ ≤ 4) are presented. Grey cells indicate the significance of parameter estimates in supported models based on 95 % confidence intervals.

| **Model** | **RD** | **RD^2^** | **MT** | **Temp.** | **RD:MT** | **RD:Temp.** | **RD^2^:MT** | **RD^2^:Temp.** | **MT:Temp.** | **RD:MT:**  **Temp** | **RD^2^:MT:**  **Temp** | **k** | **AICc** | **ΔAICc** | ***w_i_*** |
| --- | --- | --- | --- | --- | --- | --- | --- | --- | --- | --- | --- | --- | --- | --- | --- |
| 96 | - | + | + | + | + |  | - |  |  |  |  | 9 | -104.5 | 0.00 | 0.343 |
| 88 | - | + | + |  | + |  | - |  |  |  |  | 8 | -102.9 | 1.63 | 0.152 |
| 224 | - | + | + | + | + |  | - | + |  |  |  | 10 | -102.7 | 1.84 | 0.137 |
| 128 | - | + | + | + | + | + | - |  |  |  |  | 10 | -102.6 | 1.93 | 0.131 |
| 352 | - | + | + | + | + |  | - |  | + |  |  | 10 | -101.3 | 3.20 | 0.069 |

Table S4. Parameter estimates from the analysis of the variability of the abundance of dead adult hosts in experimental microcosms.

Estimates of fixed effects and their 95% confidence intervals (CIs) are differences (in number of dead adult hosts) from the intercept (i.e., “constant temperature - H microcosms” treatment group). Variance differed between microcosm types (Bartlett’s test for microcosm type: K^2^ = 5.56, df = 1, P = 0.02). Estimates of the variance function and their 95% CIs compare to the variance of the intercept, here “H microcosms”, which is set to 1. Significant estimates (i.e., different from 0 for fixed effects and from 1 for the variance function) are in bold. “Model” indicates which supported model (Table S3) was used to obtain parameter estimates. “RD” refers to resource degradation, “MT” to microcosm type, “H-P” to the host-parasitoid microcosms and “Temp.” to temperature treatments.

| **Model** | **Fixed effects** | **Estimates** | **95% CIs** |
| --- | --- | --- | --- |
| **96** | **RD** | **-0.01** | **[-0.015, -0.006]** |
| **96** | **RD^2^** | **0.00008** | **[0.00002, 0.0001]** |
| 96 | MT |  |  |
|  | H-P | 0.03 | [-0.06, 0.11] |
| **96** | **Temp.** |  |  |
|  | **Variable** | **0.04** | **[0.0008, 0.08]** |
| **96** | **RD:MT** |  |  |
|  | **H-P** | **0.01** | **[0.005, 0.016]** |
| 128 | RD:Temp. |  |  |
|  | Variable | 0.0007 | [-0.0007, 0.002] |
| **96** | **RD^2^:MT** |  |  |
|  | **H-P** | **-0.0001** | **[-0.0002, -0.00005]** |
| 224 | RD^2^:Temp. |  |  |
|  | Variable | 0.000009 | [-0.000008, 0.00003] |
| 352 | MT:Temp. |  |  |
|  | H-P:Variable | 0.004 | [-0.08, 0.09] |
| **Model** | **Variance function** | **Estimates** | **95% CIs** |
| **96** | **MT** |  |  |
|  | **H-P** | **0.61** | **[0.41, 0.91]** |

Table S5. AIC_c_ table from the analysis of the mean abundance of dead adult parasitoids in experimental microcosms.

The “+” and “-” symbols indicate the fixed effect(s) included in each model and the direction of the effect (i.e., positive or negative) on mean abundance of dead adult parasitoids. The model, number of parameters (*k*), AIC_c_ scores, model support (ΔAIC_c_) and model weights (*w_i_*) are provided for each model. “RD” refers to resource degradation, “MT” to microcosm type and “Temp.” to temperature treatments. Only the best supported models (ΔAIC_c_ ≤ 4) are presented. Grey cells indicate the significance of parameter estimates in supported models based on 95 % confidence intervals.

| **Model** | **RD** | **RD^2^** | **Temp.** | **RD:Temp.** | **RD^2^:Temp.** | **k** | **AICc** | **ΔAICc** | ***w_i_*** |
| --- | --- | --- | --- | --- | --- | --- | --- | --- | --- |
| 5 |  |  | - |  |  | 4 | 143.9 | 0.00 | 0.384 |
| 6 | - |  | - |  |  | 5 | 145.3 | 1.47 | 0.184 |
| 1 |  |  |  |  |  | 3 | 145.4 | 1.51 | 0.181 |
| 2 | - |  |  |  |  | 4 | 146.9 | 3.05 | 0.083 |
| 14 | - |  | - | + |  | 6 | 147.2 | 3.29 | 0.074 |

Table S6. Parameter estimates from the analysis of the mean abundance of dead adult parasitoids in experimental microcosms.

Estimates of fixed effects and their 95% confidence intervals (CIs) are differences (in number of dead adult parasitoids) from the intercept (i.e., “constant temperature” treatment group). Variance differed between temperature treatments (Bartlett’s test: K^2^ = 6.47, df = 1, P = 0.01). Estimates of the variance function and their 95% CIs compare to the variance of the intercept, here “H microcosms”, which is set to 1. Significant estimates (i.e., different from 0 for fixed effects and from 1 for the variance function) are in bold. Significant estimates are in bold. “Model” indicates which supported model (Table S5) was used to obtain parameter estimates. “RD” refers to resource degradation and “Temp.” to temperature treatments.

| **Model** | **Fixed effects** | **Estimates** | **95% CIs** |
| --- | --- | --- | --- |
| **6** | RD | -0.03 | [-0.08, 0.02] |
| **5** | **Temp.** |  |  |
|  | **Variable** | **-4.49** | **[-8.78, -0.20]** |
| 14 | RD:Temp. |  |  |
|  | Variable | 0.09 | [-0.06, 0.23] |
| **Model** | **Variance function** | **Estimates** | **95% CIs** |
| **5** | **Temp.** |  |  |
|  | **Variable** | **0.37** | **[0.21, 0.66]** |

Table S7. AIC_c_ table from the analysis of variability of the abundance of dead adult parasitoids in experimental microcosms.

The “+” and “-” symbols indicate the fixed effect(s) included in each model and the direction of the effect (i.e., positive or negative) on the variability of the abundance of dead adult parasitoids. The model, number of parameters (*k*), AIC_c_ scores, model support (ΔAIC_c_) and model weights (*w_i_*) are provided for each model. “RD” refers to resource degradation, “MT” to microcosm type and “Temp.” to temperature treatments. Only the best supported models (ΔAIC_c_ ≤ 4) are presented. Grey cells indicate the significance of parameter estimates in supported models based on 95 % confidence intervals.

| **Model** | **RD** | **RD^2^** | **Temp.** | **RD:Temp.** | **RD^2^:Temp.** | **k** | **AICc** | **ΔAICc** | ***w_i_*** |
| --- | --- | --- | --- | --- | --- | --- | --- | --- | --- |
| 4 | + | - |  |  |  | 4 | -93.8 | 0.00 | 0.297 |
| 8 | + | - | - |  |  | 5 | -93.6 | 0.18 | 0.271 |
| 24 | + | - | - |  | + | 6 | -93.2 | 0.62 | 0.218 |
| 16 | + | - | - | + |  | 6 | -92.8 | 1.00 | 0.180 |

Table S8. Parameter estimates from the analysis of the variability of the abundance of dead adult parasitoids in experimental microcosms.

Estimates of fixed effects and their 95% confidence intervals (CIs) are differences (in number of dead adult parasitoids) from the intercept (i.e., “0 % MC”). Significant estimates are in bold. “Model” indicates which supported model (Table S7) was used to obtain parameter estimates. “RD” refers to resource degradation and “Temp.” to temperature treatments.

| **Model** | **Fixed effects** | **Estimates** | **95% CIs** |
| --- | --- | --- | --- |
| **4** | **RD** | **0.002** | **[0.00002, 0.03]** |
| **4** | **RD^2^** | **-0.00004** | **[-0.00006, -0.00002]** |
| 8 | Temp. |  |  |
|  | Variable | -0.02^*^ | [-0.04, 0.005] |
| 16 | RD:Temp. |  |  |
|  | Variable | 0.0006 | [-0.0002, 0.001] |
| 24 | RD^2^:Temp. |  |  |
|  | Variable | 0.000008 | [-0.000002, 0.00002] |

^*^ The negative effect of fluctuating temperatures on the variability of the abundance of adult parasitoids was significant in two of the four supported models which also included a non-significant interaction term between resource degradation and temperature (see Table S7).

# Appendix S7 – Contribution of direct and indirect effects of resource degradation and temperature variation on host and parasitoid dynamics

In host populations, resource degradation and temperature variation could affect population dynamics through direct and indirect effects (i.e., through the trophic interaction with its parasitoid) while in parasitoid populations, resource degradation could only affect population dynamics through its host (i.e., indirect effects) and temperature variation could have both direct and indirect effects.

In the host data, ‘host-alone’ (H) and ‘host-parasitoid’ (H-P) microcosms were used to estimate the relative contribution of direct (in H and H-P microcosms) and indirect (only in H-P microcosms) effects of resource degradation and temperature variation on host dynamics. To do so, we estimated the explanatory power of resource degradation (rd), temperature variation (t), microcosm type (mt) and their first- and second-order interactions following Brooks *et al.* (2016), with the indirect effects of resource degradation and temperature variation captured by the interaction terms with microcosm type, which measure differences in responses to experimental treatments between H and H-P microcosms. The explanatory power of each term was obtained by comparing the proportion of null deviance explained by 7 models including the following subsets of explanatory variables and first- and second-order interactions: model 1: Y~rd+t+mt+rd:t+rd:mt+t:mt+rd:t:mt, model 2: Y~rd+t+rd:t, model 3: Y~rd+mt+rd:mt, model 4: Y~t+mt+t:mt, model 5: Y~rd, model 6: Y~t and model 7: Y~mt (Table S1 and Fig. S1).

In the parasitoid data, only the indirect effects of temperature variation transmitted through an interaction with resource degradation can be estimated. However, the main effects of temperature variation in statistical models of parasitoid population data could encompass the part of indirect effects of temperature variation transmitted through its host independently of resource degradation, if significant. Temperature variation had a very small impact on the host populations, independently of resource degradation (proportion of ≤ 0.01 of the explanatory power in host data; see the main effect of temperature variation in Table S1 and Fig. S1) and should therefore have had a limited indirect impact on parasitoid populations independently of resource degradation. We refer to the contribution of the indirect effects of temperature variation in parasitoid data transmitted through an interaction with resource degradation as the ‘minimum relative contribution’ for accuracy.

Table S1. Contribution of direct and indirect effects of resource degradation and temperature variation on host temporal change in abundance, overall mean abundance and variability in abundance and on temporal change in normalized amplitudes.

Direct effects of resource degradation (RD) and temperature variation (Temp.) are in bold and indirect effects through the trophic interaction with the parasitoid are in italic (interaction terms with microcosm type (MT)). ‘Expl. power’ refers to the proportion of explanatory power of each variable and ‘Relative contrib.’ to the relative contribution of resource degradation (RD) or temperature variation (Temp.) terms to the total explanatory power of resource degradation or temperature variation. Letters between brackets refers to the name of each explanatory variables in Fig. S1.

|  | **Temporal change in abundance** | | | **Overall mean abundance** | | | **Variability of abundance** | | | **Temporal change in amplitudes** | | |
| --- | --- | --- | --- | --- | --- | --- | --- | --- | --- | --- | --- | --- |
| **Explanatory variable** | **Expl. power (proportion)** | **Relative contrib. of RD terms (%)** | **Relative contrib. of Temp. terms (%)** | **Expl. power (proportion)** | **Relative contrib. of RD terms**  **(%)** | **Relative contrib. of Temp. terms**  **(%)** | **Expl. power (proportion)** | **Relative contrib. of RD terms (%)** | **Relative contrib. of Temp. terms (%)** | **Expl. power (proportion)** | **Relative contrib. of RD terms (%)** | **Relative contrib. of Temp. terms (%)** |
| **RD** | **0.15** | **64.3** |  | **0.17** | **80.0** |  | **0.42** | **81.6** |  | **0.32** | **68.0** |  |
| **Temp.** | **0.002** |  | **4.7** | **0.005** |  | **47.5** | **0.02** |  | **51.8** | **0.01** |  | **19.0** |
| **MT** | 0.23 |  |  | **0.70** |  |  | **0.28** |  |  | **0.07** |  |  |
| **RD:Temp.** | **0.01** | **5.4** | **36.8** | **0.003** | **1.4** | **31.2** | **0.004** | **0.8** | **13.5** | **0.03** | **6.0** | **56.0** |
| **RD:MT** | *0.06* | *25.9* |  | *0.04* | *17.6* |  | *0.08* | *15.6* |  | *0.11* | *23.8* |  |
| **Temp.:MT** | *0.01* |  | *28.6* | *0.000005* |  | *0.05* | *0.00004* |  | *0.1* | *0.002* |  | *4.8* |
| **RD:Temp.:MT** | *0.01* | *4.4* | *29.9* | *0.002* | *1.0* | *21.3* | *0.01* | *2.1* | *34.6* | *0.01* | *2.2* | *20.2* |
| **Total** | 0.48 |  |  | *0.91* |  |  | *0.81* |  |  | *0.56* |  |  |

Table S2. Contribution of direct and indirect effects of resource degradation and temperature variation on parasitoid temporal change in abundance, overall mean abundance and variability in abundance.

Direct effects of temperature variation (Temp.) are in bold and indirect effects of resource degradation (RD) and temperature variation^†^ are in italic (interaction term between resource degradation and temperature variation). ‘Expl. power’ refers to the proportion of explanatory power of each variable and ‘Relative contrib.’ to the relative contribution of resource degradation (RD) or temperature variation (Temp.) terms to the total explanatory power of resource degradation or temperature variation.

|  | **Temporal change in abundance** | | | **Overall mean abundance** | | | **Variability of abundance** | | |
| --- | --- | --- | --- | --- | --- | --- | --- | --- | --- |
| **Explanatory variables** | **Expl. power (proportion)** | **Relative contrib of RD terms (%)** | **Relative contrib. of Temp. terms (%)** | **Expl. power (proportion)** | **Relative contrib of RD terms (%)** | **Relative contrib. of Temp. terms (%)** | **Expl. power (proportion)** | **Relative contrib of RD terms (%)** | **Relative contrib. of Temp. terms (%)** |
| **RD** | *0.06* | *75.1* |  | *0.14* | *49.1* |  | *0.64* | *94.2* |  |
| **Temp.** | **0.01** |  | **43.6** | **0.18** |  | **55.2** | **0.04** |  | **52.2** |
| **RD:Temp.** | *0.02* | *24.9* | *56.4* | *0.14* | *50.9* | *44.8* | *0.04* | *5.8* | *47.8* |
| **Total** | 0.09 |  |  | 0.46 |  |  | 0.72 |  |  |

^†^ It is to be noted that only the indirect effects of temperature variation transmitted through an interaction with resource degradation can be estimated in parasitoid population data. This is because the main effects of temperature variation in these models could encompass the part of indirect effects of temperature variation transmitted through its host independently of resource degradation, if significant. However, temperature variation had a very small impact on host populations independently of resource degradation (proportion of ≤ 0.01 of the explanatory power in host data; see the main effect of temperature variation in Table S1 and Fig. S1) and should therefore have had a limited indirect impact on parasitoid populations independently of resource degradation.

**
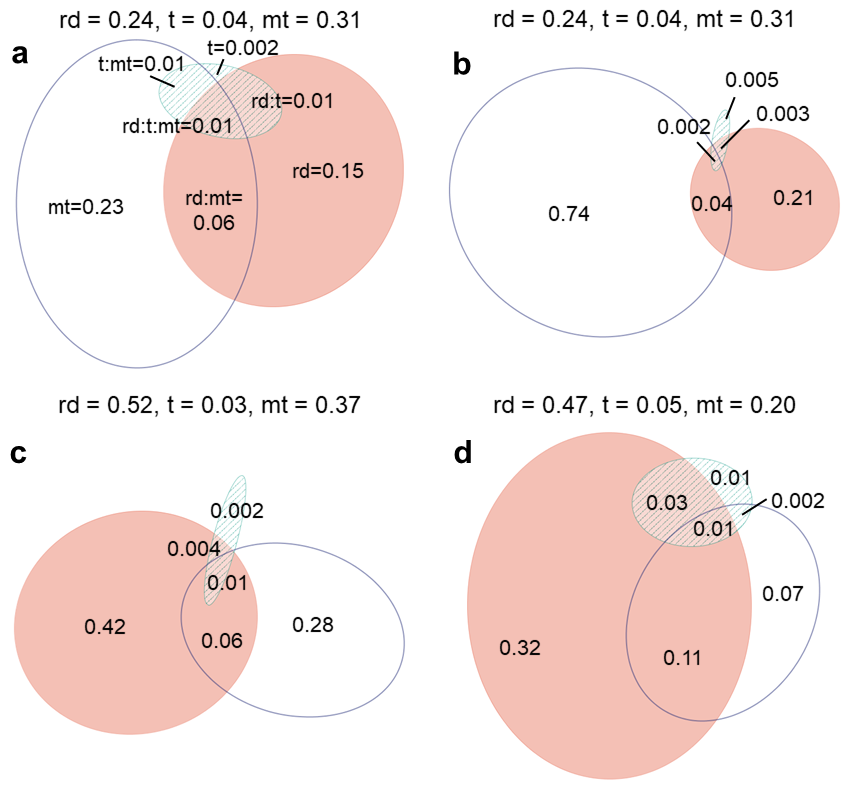
**

**Figure S1.** Area-proportional Venn diagrams (Micallef & Rodgers 2014) representing the overlapping proportion of explanatory power of resource degradation (rd, pink ellipses), temperature variation (t, green striped ellipses) and microcosm type (mt, open ellipses) for the temporal change in (a) host abundance, (b) overall mean host abundance (top right panel), (c) overall variability in host abundance and (d) temporal change in host normalized amplitudes (bottom right). Overlapping areas with microcosm type represent the indirect effects of resource degradation and temperature variation on host populations.

# References

Barton, K. (2018) MuMIn: Multi-Model Inference. <https://CRAN.R-project.org/package=MuMIn>.

Begon, M., Sait, S.M. & Thompson, D.J. (1996) Predator-prey cycles with period shifts between two- and three-species systems. *Nature,* **381,** 311-315.

Briggs, C.J., Sait, S.M., Begon, M., Thompson, D.J. & Godfray, H.C.J. (2000) What causes generation cycles in populations of stored-product moths? *Journal of Animal Ecology,* **69,** 352-366.

Brooks, M.E., Mugabo, M., Rodgers, G.M., Benton, T.G. & Ozgul, A. (2016) How well can body size represent effects of the environment on demographic rates? Disentangling correlated explanatory variables. *Journal of Animal Ecology,* **85,** 318-328. https://doi.org/10.1111/1365-2656.12465

Burnham, K.P. & Anderson, D.R. (2002) *Model selection and multimodel inference. A practical information-theoretic approach*. Springer, New York.

Cryer, J.D. & Chan, K.-S. (2009) *Time series analysis with applications in R,* Second edition edn. Springer.

Fussmann, K.E., Schwarzmuller, F., Brose, U., Jousset, A. & Rall, B.C. (2014) Ecological stability in response to warming. *Nature Climate Change,* **4,** 206-210. https://doi.org/10.1038/nclimate2134

Kim, D. & Oh, H.-S. (2009) EMD: a package for Empirical Mode Decomposition and Hilbert Spectrum. *The R Journal*, pp. 40-46. <https://cran.r-project.org/web/packages/EMD/index.html>.

Micallef, L. & Rodgers, P. (2014) eulerAPE: Drawing Area-Proportional 3-Venn Diagrams Using Ellipses. *Plos One,* **9**. https://doi.org/10.1371/journal.pone.0101717

Sait, S.M., Begon, M. & Thompson, D.J. (1994) Long-term population-dynamics of the Indian meal moth *Plodia-interpunctella* and its granulosis-virus. *Journal of Animal Ecology,* **63,** 861-870.
